# Harnessing microRNA regulatory networks for engineering climate resilience and nutritional enhancement in millets

**DOI:** 10.1007/s44154-026-00332-2

**Published:** 2026-08-10

**Authors:** Kasanaboina Krishna, Ephrem Habyarimana, Harsha Rayudu Jamedar, Ishwarya Lakshmi VG, Sonal Chavan, B. V. Vara Prasad, Y. Chandra Mohan, Bandela Edukondalu, Stanislaus Antony Ceasar

**Affiliations:** 1 International Crop Research Institute for the Semi-Arid Tropics (ICRISAT), Patancheru, Telangana 502324 India; 2 Indian Council of Agricultural Research (ICAR), Indian Institute of Oilseeds Research (IIOR), Rajendranagar, Hyderabad, Telangana 500030 India; 3 Texas A&M AgriLife Research Center, Beaumont, TX 77713 USA; 4 Professor Jayashankar, College of Agriculture, Rajendranagar (PJTAU), Telangana Agricultural University, Hyderabad, Telangana 500030 India; 5 Department of Biotechnology, Karunya Institute of Technology and Sciences, Tamil Nadu, Coimbatore, 641114 India

**Keywords:** Millets, MicroRNA, Abiotic stress, Drought tolerance, Salinity, Nutrient biofortification, Degradome, Genome editing

## Abstract

Millets are increasingly promoted as climate-resilient nutri-cereals that can stabilize production and support nutritious diets under drought, heat, salinity, and low-input farming. MicroRNAs (miRNAs) provide a powerful post-transcriptional regulatory layer that regulates stress signaling, crop development, nutrient homeostasis, and grain filling by targeting key transcription factors and transporter networks. Millet miRNA research remains at an early developmental stage. To date, most studies have focused predominantly on miRNA identification and expression profiling, while rigorous functional characterization and mechanistic dissection of regulatory networks are still limited. Moreover, integration of miRNA dynamics with genotype (G) × environment (E) interactions, particularly under field-relevant stress conditions, remains insufficiently explored. In this review, we synthesize stress- and nutrition-associated miRNAs reported across major cereals, highlighting conserved regulatory modules, and summarize emerging millet-specific candidates linked to drought, salinity, grain quality, and micronutrient accumulation. We critically evaluate methodological gaps, such as incomplete degradome support, inconsistent phenotyping, and limited grain ionomics, that constrain translational application to breeding. Finally, we propose a practical roadmap that combines tissue- and stage-resolved miRNA atlases, target validation using degradome sequencing, RNA Ligase-Mediated Rapid Amplification of cDNA Ends (RLM-RACE), and reporter assays, together with functional intervention platforms such as Short Tandem Target Mimic (STTM), artificial miRNAs (amiRNAs), and CRISPR-mediated editing of miRNA loci or target sites, all integrated with marker-enabled selection and genomic prediction. Advancing from descriptive miRNA catalogues toward experimentally validated regulatory networks will enable miRNA-based breeding and genome editing for climate-smart, micronutrient-dense millet cultivars.

## Introduction

Millets are a broad category of small-seeded cereals that are valued for their high nutritional content and crucial function in climate-resilient agriculture. Millets are generally classified into major millets [sorghum (*Sorghum bicolor*), pearl millet (*Cenchrus americanus*), and finger millet (*Eleusine coracana*)], minor millets [foxtail millet (*Setaria italica*)*,* proso millet (*Panicum miliaceum*)*,* kodo millet (*Paspalum scrobiculatum*)*,* barnyard millet (*Echinochloa frumentacea*)*,* little millet (*Panicum sumatrense*) and browntop millet (*Urochloa ramose*)]. Millets are highly adaptable agronomically, as they can grow on marginal soils, need little water, and can withstand extended drought (Mukherjee et al. 2025). As a result, they provide more consistent yields in areas where major cereals often fail to grow. In addition to promoting food and nutritional security, their production boosts biodiversity, fortifies farmer livelihoods, and increases climate resilience (Kumar et al. 2024). Nutritionally rich in protein, essential amino acids, minerals, vitamins, dietary fiber, and antioxidants, these “nutri-cereals” are gluten-free and have a low glycemic index, making them suitable for both general health and specialized diets (Sharma et al. 2025; Sharma et al. 2021; Shukla et al. 2025). Despite these benefits, millets are still viewed as "orphan crops" in international agriculture because of the prevalence of maize, rice, and wheat compared to millets (Wilson and VanBuren 2022). In addition, millets are often underrepresented in modern genetic and genomic research when compared to staple cereal grains (Yang et al. 2025). However, the increasing threat of climate change and the rising prevalence of malnutrition highlight the urgency of crop diversification (Mihrete and Mihretu 2025). Incorporating millets into farming systems offers a powerful strategy to diversify diets, reduce ecological pressure, and ensure economic stability for smallholder farmers. As C4 plants, millets exhibit high photosynthetic efficiency, rapid growth cycles, superior dry matter production, and remarkable tolerance to heat and drought, ensuring consistent yields under adverse conditions (Yadav et al. 2024).

Climate change exposes crops to persistent abiotic challenges, such as salinity, drought, and high temperature, as well as biotic pressures, like diseases and pests. These variables, individually or collectively, significantly hinder growth and production by affecting morphological, physiological, and molecular processes (Zandalinas and Mittler 2022). A key challenge in crop improvement is that enhancing stress tolerance often compromises other agronomic traits, such as yield or growth factors. Regulatory genes, particularly those enabling precise control, offer a promising solution. Among modern tools, microRNAs (miRNAs) have attracted considerable attention as contemporary tools for their involvement in stress adaptation.

**Fig. 1:**
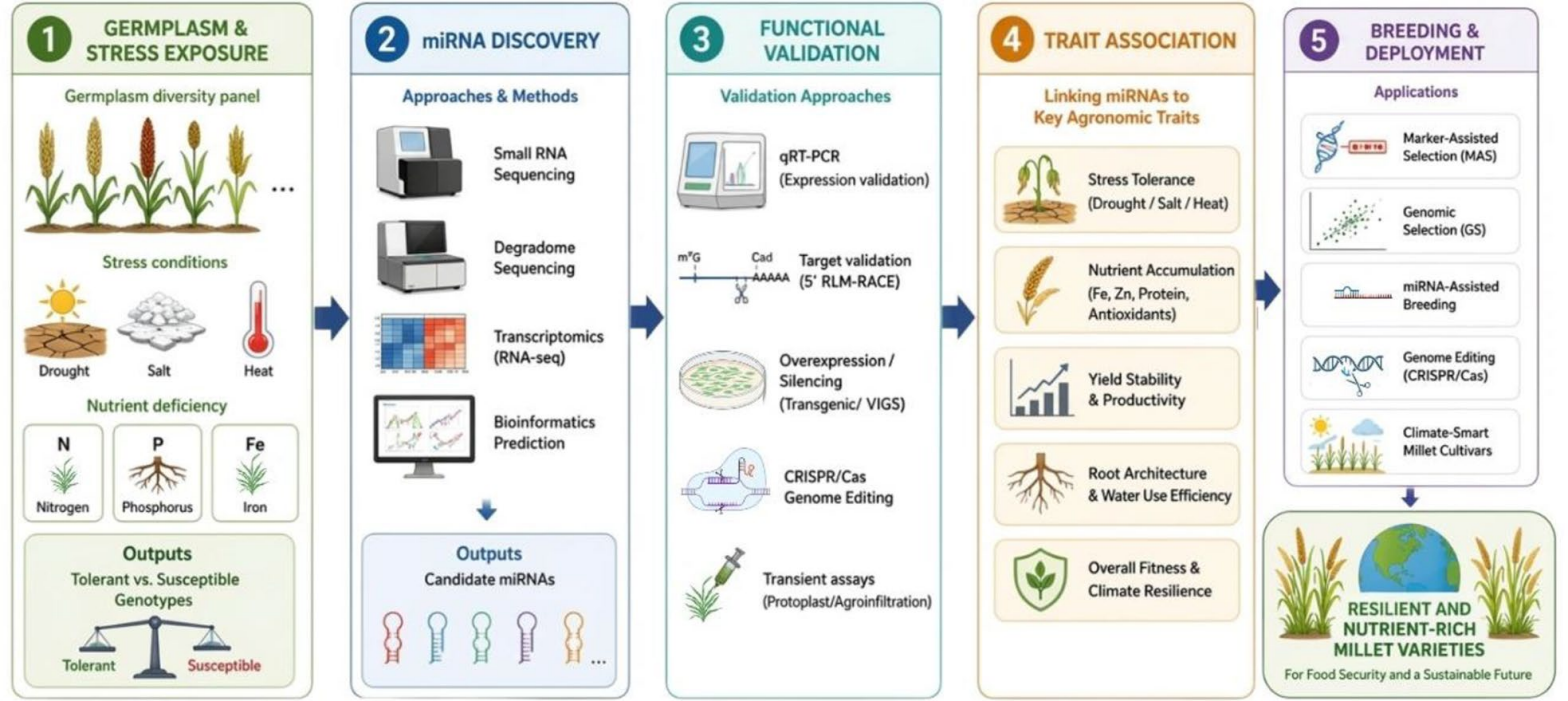
Workflow illustrating the translational pipeline from millet miRNA discovery to crop improvement. The framework includes germplasm screening under abiotic and nutrient stresses, miRNA identification using sequencing and bioinformatics approaches, functional validation through molecular and genome-editing tools, trait association analyses, and integration into breeding programs for developing climate-resilient and nutrient-rich millet cultivars

The miRNAs are short, single-stranded, non-coding RNA molecules, typically 21–24 nucleotides in length, that primarily function in post-transcriptional gene regulation (Dong et al. 2022). Their mature forms are highly conserved, a feature that has drawn significant attention to their roles in plant growth, development, and responses to stress across diverse species. By binding to target genes, miRNAs modulate gene expression and influence a wide range of biological processes, including development, metabolism, and transcriptional control through interactions with transcription factors (TFs) (Gangadhar et al. 2021). The interaction between a miRNA and its corresponding mRNA target is crucial, as it directly influences the variation in gene expression. Gene regulation by miRNAs occurs through several mechanisms, such as mRNA cleavage, translational repression, chromatin remodeling, and epigenetic modification (Kumar et al. 2018). A single miRNA can target multiple genes within the same cellular signaling pathway (Alptekin et al. 2017). Although miRNA loci constitute only a very small fraction of plant genomes, miRNAs are predicted to regulate a substantial proportion of protein-coding genes and play critical roles in plant growth, development, and stress responses (Zhang et al. 2007). Multiple studies have indicated that environmental alterations might modify the miRNA expression profile, consequently modulating plant stress responses (Bej and Basak 2014). Recently, the discovery and analysis of miRNAs have become a major area of interest in plant molecular biology. Progress in genomics, encompassing microarrays and next-generation sequencing (NGS), has elucidated multiple stress-associated protein-coding genes (Varshney et al. 2020) but uncertainties persist regarding their regulation. Recent findings underscore miRNAs as small regulatory RNAs that function as widespread post-transcriptional regulators, influencing plant growth, development, and stress responses (Khraiwesh et al. 2012). Despite the agronomic resilience and nutritional value of millets, the regulatory mechanisms that connect stress adaptation, nutrient use efficiency, grain development, and micronutrient accumulation remain incompletely understood. In particular, miRNA-mediated regulatory networks in millets are far less developed than those in rice, wheat, maize, and Arabidopsis. Available millet studies are still dominated by computational prediction, small RNA sequencing, and expression profiling, whereas experimentally validated miRNA target interactions, degradome-supported cleavage evidence, tissue- and stage-specific regulatory maps, and field-relevant genotype × environment interpretation remain limited. Therefore, this review lies not in providing a general account of millet biology or plant miRNA function but in organizing the available evidence into a millet focused regulatory framework. Specifically, this review distinguishes conserved grass-wide miRNA modules from emerging millet-specific candidates, evaluates their relevance to drought, heat, salinity, root architecture, grain quality, and micronutrient accumulation, and identifies where evidence is strong, preliminary, or only inferred from model systems. By integrating current millet-specific findings with validated cereal miRNA networks, we propose a practical roadmap for moving millet miRNA research from descriptive cataloguing toward functional validation, marker-enabled breeding, genomic prediction, and genome editing-based improvement of climate-resilient and nutrient-dense millet cultivars (Fig. 1).

## Review framework and literature-search strategy

A systematic literature search strategy was employed to identify relevant studies on miRNA-mediated regulation of climate resilience, nutrient use efficiency, and nutritional traits in millets. Comprehensive searches were performed in Web of Science, Scopus, PubMed, Google Scholar, ScienceDirect, SpringerLink, NCBI resources, miRBase, and other plant small RNA databases. The search utilized combinations of keywords including “millet microRNA,” “millet miRNA,” “small RNA sequencing in millets,” “miRNA drought tolerance,” “miRNA salinity tolerance,” “miRNA heat stress,” “miRNA nutrient stress,” “millet grain quality,” “millet nutritional traits,” “degradome sequencing,” “RLM-RACE,” “artificial microRNA,” “STTM,” and “genome editing”. Millet-specific studies were given priority, particularly those reporting miRNA identification, expression profiling, predicted or validated targets, degradome-supported cleavage, or experimental functional validation.

## Climate-resilient properties of millets

The concise biological context for understanding why miRNA regulation is particularly relevant in millets, rather than providing an extensive description of millet stress physiology, places the emphasis on traits that are likely to be under regulatory control, such as root architecture, osmotic adjustment, antioxidant defense, flowering plasticity, nutrient uptake, and grain filling. These traits provide the physiological entry points through which miRNA target modules may contribute to climate resilience. Current agricultural production is increasingly constrained by climate change, particularly due to rising incidences of drought and heat stress, which together cause greater yield losses than most other abiotic stresses. Climate models predict that drought will continue to be a major threat to global food production (Simmons et al. 2020). Drought stress affects multiple physiological and biochemical processes in millet crops, including pigment stability, membrane integrity, osmotic balance, photosynthetic efficiency, water regulation, yield potential, and even root-associated microbiome composition (Veach et al. 2020). Unique morphological and structural traits, such as compact stature, extensive and deep-rooted systems, thicker cell walls, and smaller leaf areas, contribute significantly to their stress tolerance (Bandyopadhyay et al. 2017). Furthermore, the C4 photosynthetic pathway provides additional resilience by facilitating efficient carbon fixation, flexible biomass partitioning, and reduced water loss per unit leaf area, thereby ensuring survival under heat and water-deficit conditions (Table 1) (Lundgren et al. 2019).

**Table 1:** Climate-resilient properties of different millet species. The table summarizes the climate resilient properties of various millets with references

S. No	Millet	Stress tolerance	Rainfall requirement (mm)	Salinity threshold (dS m⁻^1^)	References
1	Sorghum	Drought tolerant	400–500	~ 7	(Borrell et al. 2024)
2	Pearl millet	Drought and heat	250–350	11–12	(Yadav et al. 2020)
3	Barnyard millet	Drought and waterlogging	350–400	11–12	(Mohanapriya et al. 2024)
4	Finger millet	Drought and cold tolerant	400–1000	~ 6	(Mirza and Marla 2019)
5	Foxtail millet	Drought tolerant	300–400	~ 6	(Al-Khayri et al. 2019; Ceasar and Baker 2025)
6	Kodo millet	Drought tolerant	400–500	3–5	(Ravikesavan et al. 2022)
7	Little millet	Drought and waterlogging	300–500	~ 6	(Mushtaq et al. 2021; Raj et al. 2025)
8	Proso millet	Drought, salinity and heat	300–400	~ 8–10	(Samineni et al. 2025; Zou et al. 2019a, b)
9	Browntop millet	Drought tolerant	130–340	~ 5–6	(Bhavani et al. 2024; Mushtaq et al. 2021)
10	Tef	Drought and waterlogging	300–500	~ 6	(Barretto et al. 2021; Ceasar and Baker 2025)
11	Fonio	Drought and waterlogging	700–1000	~ 5–6	(Ibrahim Bio Yerima and Achigan‐Dako 2021)

Rainfall values represent approximate crop requirement or cultivation ranges reported in the cited sources and may vary with genotype, soil type, crop duration, and production environment. Salinity values are reported according to the measurement basis used in the original studies, such as soil electrical conductivity, irrigation water electrical conductivity, or experimental salinity treatment. Therefore, these values should be interpreted as indicative ranges rather than fixed tolerance thresholds

High temperatures exacerbate crop stress by interfering with the electron transport chain, affecting photosystem II (PSII) performance, and increasing reactive oxygen species (ROS) formation (Ray et al. 2019). The majority of millets, in contrast to rice and maize, have developed various acclimatization, avoidance, and adaptation strategies to mitigate heat stress. Their resilience is increased by the synthesis of osmoprotectants, heat-shock proteins (HSP), transcription factors, ion transporters, and signaling molecules (Kasanaboina et al. 2025; Mukesh Sankar et al. 2021). Compared to maize, millets like sorghum are more sustainable crop options because they require less water and fertilizer, can be grown in marginal areas with poor soil fertility and structure, are more resilient to high radiation and insufficient and erratic rainfall, have shorter growth cycles, and yield more consistently (Mundia et al. 2019). While certain plants perform better in more acidic soils, others perform better in more alkaline ones. When pearl millet is grown in oxisols as opposed to ultisols, higher yields are seen. Although there has been some evidence of a production penalty, pearl millet survived well even in situations where the soil was relatively acidic (Flores et al. 1991). Moreover, millets with strong antioxidant defenses against oxidative damage brought on by several abiotic stressors include finger millet and barnyard millet (Tiwari et al. 2020). The exceptional adaptability of browntop millet allows it to flourish on slightly acidic or saline soils (Bhavani et al. 2024). It is an excellent option for restoring degraded soils and raising agricultural production due to its hardiness (Abhigna 2024). In marginal, rainfed agricultural systems with irregular and low rainfall, proso millet has adapted effectively (Samineni et al. 2025). Tef has historically been grown as a low-risk crop, especially in areas that are vulnerable to moisture stress, where it provides a reliable substitute in the event that other cereal crops fail. It can also be cultivated in poorly drained soils that are unsuitable for crops like maize and wheat, and it has a moderate tolerance to drought (Assefa et al. 2017; Cannarozzi and Tadele 2022; Chanyalew et al. 2019). Fonio grows well in dryland agricultural systems because it is very tolerant to drought and can adapt to nutrient-poor soils (Tabassum et al. 2024). Finger millet showed stable emergence and yield even under low-input circumstances, in comparison to maize and sorghum, when tested under different fertilization rates and planting dates (Rurinda et al. 2014).

## Nutritional benefits of millets

Millets are increasingly recognized as nutri-cereals (Lydia Pramitha et al. 2023) because of their favorable grain composition, including appreciable levels of carbohydrates, dietary fiber, protein, essential amino acids, minerals, vitamins, and phytochemicals (Hassan et al. 2021). As naturally gluten-free cereals, millets can be suitable for gluten-free diets, particularly when processed under conditions that avoid cross-contamination with gluten-containing grains (Rai et al. 2018). Compared with refined rice and wheat-based diets, several millet grains contain higher levels of dietary fiber, minerals, and antioxidant compounds, making them useful components of diversified and nutrient-sensitive food systems (Sharma et al. 2025). Finger millet is particularly known for its high calcium content (Maharajan et al. 2021; Lydia Pramitha et al. 2023), whereas pearl millet is valued for its iron and zinc content (Anitha et al. 2021a, b). Kodo millet contains high dietary fiber and phenolic compounds; barnyard millet is associated with magnesium, phosphorus, dietary fiber, and a low glycemic index (Sanjay Kumar et al. 2024), and fonio is valued for sulfur-containing amino acids such as methionine and cysteine (Lydia Pramitha et al. 2021). Browntop millet has also been reported to contain high dietary fiber and useful mineral constituents, including magnesium. These nutritional attributes support the inclusion of millets as part of cereal diversification strategies, especially in regions where diets are dominated by a narrow range of staple grains. Although pearl millet and finger millet may contain substantial amounts of iron, zinc, and calcium, the absorption of these minerals is influenced by anti-nutritional factors such as phytates, polyphenols, tannins, and dietary inhibitors or enhancers. Processing methods such as soaking, germination, fermentation, malting, decortication, and cooking can modify these compounds and thereby influence nutrient bioaccessibility and bioavailability. Therefore, claims related to iron, zinc, calcium, or protein benefits should be interpreted not only on the basis of grain composition but also in relation to processing method, meal composition, and human absorption studies. Dietary intervention studies provide a separate level of evidence from compositional analysis. For example, finger millet porridge consumption has been associated with improved hemoglobin levels in schoolgirls (Karkada et al. 2019), while supplementation with a multi-millet mix has been reported to improve selected anthropometric indices in students (Anitha et al. 2024). Such studies indicate the potential nutritional contribution of millet-based foods under specific dietary and population contexts.

Similarly, the low glycemic index, high dietary fiber, mineral content, and antioxidant potential of some millet grains may support better metabolic health when included in balanced diets, particularly for populations requiring improved dietary quality (reviewed by Kumari et al. 2025). However, millets should be described as supportive dietary components rather than therapeutic agents. Their potential relevance to glycemic control, anemia risk reduction, gut health, and metabolic disease management depends on processing, portion size, overall diet quality, clinical status, and long-term dietary adherence. Thus, integrating millets into food systems may contribute to nutritional security and diet diversification while also supporting sustainable and resource-efficient agriculture (Table 2).

**Table 2:** Nutritional contents of millets with their nutritional advantages and health benefits. The table summarizes the nutritional profile of various millets

Crop	CHO (g)	Protein	Crude fiber	Ca	P	Fe	Zn	Mg	Nutritional advantage	Health applications	References
Sorghum	67.7	9.9	10.2	27.6	274	3.9	1.9	133	High dietary fiber (6%)	Aids digestion and may contribute to weight management	(Tanwar et al. 2023)
Pearl millet	61.8	10.9	11.5	27.4	289	6.4	2.8	124	Highest iron and fat content, high in zinc and folic acid	Beneficial for pregnant women	(Gowda et al. 2022)
Finger millet	72.6	7.7	3.6	344	250	6.3	2.3	130	High in calcium, phosphorus, zinc, iron	Supports bone health; fights malnutrition	(Lydia Pramitha et al. 2023)
Foxtail millet	60.9	12.3	8	31	290	2.8	2.4	81	Rich in protein, fiber, and minerals but low in fat content	Supports dietary diversification and may contribute to glycemic regulation as part of a balanced diet	(Abedin et al. 2022)
Proso millet	60.9	12.5	5.2	14	206	0.8	1.4	81	High lecithin	Neural health	(Das et al. 2019)
Barnyard millet	65.5	6.2	13.6	20	280	8	3	137	High in magnesium, phosphorus, dietary fiber, niacin, low glycemic index	Supports balanced blood sugar; overall nutrient boost	(Lydia Pramitha et al. 2021)
Little millet	65.6	10.4	7.6	16.1	220	1.3	3.7	133	High polyunsaturated fatty acids (PUFA), flavonoids	Beneficial for cardiovascular health	(Indirani 2021)
Kodo millet	66.2	8.9	5.2	15.3	188	2.3	0.7	147	Highest fiber and phosphorus, high phenol content	Excellent for digestion; strong antioxidant capacity	(Deepak Taggelli and Thakur 2018)
Tef	73.1	13.3	8	180	429	7.6	3.6	184	Low glycemic index and rich in essential amino acids and fatty acids, and provide a well-balanced profile of minerals, vitamins, and their precursors	Promising food for the prevention and management of diabetes	(Habte et al. 2022)
Browntop millet	61.4	11.5	12.5	-	-	-	-	-	Low glycemic index	Helpful to individual with glucose tolerance and diabetes	(Anitha et al. 2021a, b)

Source: (Longvah et al. 2017; Dey et al. 2022)

Nutritional values are expressed on a grain dry weight basis unless otherwise stated. Values may vary depending on genotype, growing environment, grain processing, and analytical method. All units were standardized as g/100 g, mg/100 g, percentage, or other appropriate units according to the cited sources

## Current status of miRNA research in millets

miRNA research in millets is still at an early but rapidly developing stage. Compared with model plants and major cereals, the millet miRNA literature remains relatively sparse and is concentrated mainly on miRNA discovery, computational target prediction, and differential expression profiling under stress conditions. Nevertheless, these studies provide an important foundation for constructing millet-specific regulatory hypotheses. Current evidence suggests that several conserved miRNA families are associated with drought, salinity, heat, pathogen response, root development, flowering, and nutrient-related pathways in millets. However, only a limited number of reports have progressed from prediction or expression profiling to mechanistic validation of miRNA target interactions. Therefore, the key challenge is to distinguish miRNAs that are merely detected, computationally predicted, or expression correlated from those supported by degradome evidence, target validation, functional assays, or trait level confirmation. In the following sections, millet-specific evidence is synthesized according to stress response, development, grain quality, and nutritional traits, while clearly indicating where conclusions are experimentally supported and where they remain candidate hypotheses inferred from prediction, expression profiling, or evidence from other cereals and model systems.

### Drought and heat stress

The production level of millets remains low under stress conditions, despite their good adaptation to drought prone environments. Research on the mechanisms behind drought tolerance may boost millets' productivity. Chakraborty et al. (2020) used in silico approaches to identify novel 19 DE miRNAnd gene targets from the genomic resources of five millets (sorghum, pearl millet, finger millet, proso millet, and foxtail millet). The study identified approximately 84 conserved miRNAs across different millet species combinations, suggesting that several miRNA families may be evolutionarily conserved among millets. However, these predicted miRNA target relationships require experimental validation before they can be considered established functional modules (Chakraborty et al. 2020). The identification of two TAS3 genes, orthologous to Arabidopsis TAS3 genes and predicted as targets of miR390, suggests conservation of the miR390 TAS3 pathway in sorghum (Katiyar et al. 2015). The need to characterize sorghum genotypes using miRNA targets in order to produce droughttolerant cultivars was demonstrated by a varying expression pattern in the genotypes for the expression analysis of conserved miRNAs in response to drought (Hamza et al. 2016). In foxtail millet, miR394 (Geng et al. 2021) and SimiR396d (a member of the miR396 family) (Zhang et al. 2023) were reported to regulate drought tolerance. Sit-miR394 was upregulated after independent exogenous applications of methyl jasmonate, ethephon, salicylic acid, and abscisic acid. The noticeably higher germination rates and root lengths indicated a positive role of Sit-miR394 in the drought response (Geng et al. 2021). A similar observation of increased root length was noted due to overexpression of SimiR396d, enhancing drought tolerance in foxtail millet (Zhang et al. 2023). In pearl millet, a crop with the highest degree of tolerance to drought, efforts were made to identify miRNAs and reveal their role in drought stress adaptations. (Palakolanu et al. 2022) identified 61 novel Pg-miRNAs, with upregulation of Pg-miRNA targets in response to drought. They reported upregulation of Pg-miR167, Pg-miR172, Pg-miR396, Pg-miR399, Pg-miR862, Pg-miR868, Pg-miR950, Pg-miR5054, and Pg-miR7527 with direct and indirect roles in root physiology and abiotic stress responses. These miRNAs were associated with predicted targets related to root physiology and abiotic stress responses; however, their precise regulatory roles remain to be confirmed through degradome analysis, RLM-RACE, reporter assays, or functional validation. In foxtail millet, 18 drought-responsive miRNAs were identified (14 were upregulated and 4 were downregulated) and 3 novel miRNAs were predicted in response to drought (Wang et al. 2016). A study on sorghum miRNAs under drought, heat, and combined drought and heat stress reported varied miRNA regulations between drought and heat stress and had identified 80 individual miRNAs (of which 25 miRNAs were found to be differentially regulated in response to stress treatments) and 8 novel stress-responsive miRNA families (Puli et al. 2021). These studies provide valuable candidate miRNAs for drought and heat response, but most remain expression correlated or computationally predicted candidates until direct target and functional validation is performed.

### Salinity stress

Salinity, a severe abiotic stress that is expected to cause a loss of 10% of global arable land, has a significant negative impact on agriculture by reducing yields (Saleem et al. 2025). This emphasizes the need to understand the mechanisms underlying salinity tolerance. A study on pearl millet miRNAs and their targets in salinity has identified 95 salinity stress-responsive miRNAs (81 conserved miRNAs and 14 novel miRNAs) targeting 448 pearl millet genes regulating auxin responses (Shinde et al. 2020). These findings suggest that miRNA-mediated auxin regulation may be involved in pearl millet salinity response; however, the predicted miRNA target interactions require experimental validation before being considered established regulatory modules. In finger millet, tolerance to salinity and dehydration stress has conferred the role of the *Eco-miR169-EcNF-YA13* gene regulatory network. The EcNF-YA13 transcription factor, crucial for salinity and dehydration tolerance, is suppressed by Eco-miR169 in finger millet. Regulating Eco-miR169 could prevent this inhibition, enabling EcNF-YA13 to enhance abiotic stress tolerance (Rani et al. 2025). Modulation of *Eco-miR169* may therefore provide a potential route to improve abiotic stress tolerance, although further field-level and genotype specific validation would strengthen its translational value. Another study on finger millet had reported the identification of 48 conserved and 35 novel salinity-responsive miRNAs with more than tenfold upregulation of *miR160, miR162, miR169, miR894,* and *miR2914* in tolerant genotypes (Selvam et al. 2015). These miRNAs should be considered expression-correlated candidates for salinity tolerance until their targets and functional effects are experimentally confirmed.

### Biotic stress

For millets, biotic stresses can be devastating, causing huge yield losses, although millets have fewer biotic constraints compared to major cereals. A limited number of researchers have conducted studies on biotic stress-responsive miRNAs. In sorghum, a study on miRNA expression dynamics in response to anthracnose disease had identified 35 differentially expressed miRNAs (Fu et al. 2020). These miRNAs provide candidate regulators for pathogen response studies, but their direct contribution to disease resistance requires target validation and functional characterization. Research on sorghum mosaic virus on sweet sorghum reported that some sorghum-originated miRNAs can target the different genome regions of sorghum mosaic virus and regulate the defense-related host genes as well. They identified 24 miRNA targets located on the viral genome (Ghodoum Parizipour et al. 2025). Although these findings suggest possible miRNA involvement in antiviral defense, the predicted viral and host targets should be interpreted as candidate interactions until confirmed by target cleavage assays or functional experiments. Han et al. (2014) investigated miRNAs in foxtail millet using a bioinformatics approach and have identified 432 potential targets for 38 miRNA families, most of which were predicted to be involved in plant development, signal transduction, metabolic pathways, disease resistance, and environmental stress responses (Han et al. 2014).

### Plant growth and development

Plant growth and development determine millet plants’ architecture, phenology, and reproductive success, all of which are closely linked to adaptation in marginal and rainfed environments. MicroRNAs act as central post-transcriptional regulators of these developmental processes by fine-tuning transcription factors and hormone signaling components that control phase transitions, organ size, branching, and root system architecture (Table 3). In grasses, conserved miRNA–target modules governing development have been extensively characterized, and emerging studies indicate that these networks are also active in millets (Chakraborty et al. 2020; Han et al. 2014). One of the most critical developmental regulators is the miR156–SPL (Squamosa Promoter Binding-Like) module, which controls the juvenile-to-adult phase transition, tillering, panicle architecture, and flowering time in cereals. Elevated miR156 expression delays flowering and promotes vegetative branching, while reduced miR156 accelerates reproductive development. Genome-wide identification of SPL gene families and miR156 loci in foxtail millet and sorghum confirms the conservation of this regulatory module in millets, suggesting a role in regulating early flowering and life-cycle plasticity that enables drought escape (Chakraborty et al. 2020; Han et al. 2014).

**Table 3:** miRNAs identified in different developmental stages and stress conditions of millets with their evidence status

Crop	Stress	miRNA	Role/Strategy	Evidence status	Reference
Finger millet	Dehydration and salinity stress	miR169	Prevents inhibition of EcNF-YA13 gene	Experimentally supported millet specific module; retain as validated where target suppression and trait-level evidence are reported	(Rani et al. 2025)
Foxtail millet	Drought	miR394	Overexpression of Sit-miR394 affected the expression of a set of stress-related genes	Functional evidence in millet; downstream target validation remains limited	(Geng et al. 2021)
	Drought and development	miR396 (SimiR396d)	Targets *SiGRF1*, regulates root length and growth	Experimentally supported millet miRNA target module	(Zhang et al. 2023)
Finger millet	Salinity	miR160, miR162, miR169, miR894 and miR2914	Up regulation confers salinity tolerance	Expression correlated candidates; direct target and functional validation required	(Selvam et al. 2015)
Pearl millet	Drought/root development	miR167, miR172, miR396	Regulate root architecture and stress adaptation	Expression correlated candidates; regulatory roles inferred from expression and predicted targets	(Palakolanu et al. 2022)
Sorghum	Anthracnose disease	miR156, miR398, miR169	Differential expression during pathogen infection	Expression correlated candidates; functional disease resistance roles require validation	(Fu et al. 2020)
Sorghum	Viral infection/SrMV	Multiple conserved miRNAs	Target viral genome and defense genes	Predicted/target candidate evidence; antiviral function requires experimental confirmation	(Ghodoum Parizipour et al. 2025)
Foxtail millet	Growth and development	miR156	Predicted regulation of SPL genes controlling phase transition	Predicted conserved module in millet; function inferred from conserved miR156 SPL biology	(Han et al. 2014)
Foxtail millet	Growth and development	miR172	Predicted regulation of flowering and floral identity	Predicted conserved module in millet; functional validation required	(Han et al. 2014)
Multiple millets	Development & stress	miR160, miR167	Auxin mediated root and shoot development	Predicted/conserved candidate module; millet specific functional validation required	(Chakraborty et al. 2020)
Multiple millets	Leaf & biomass development	miR396	Regulation of GRF mediated organ growth	Predicted/conserved candidate module; millet specific functional validation required, except where SimiR396d–SiGRF1 has been experimentally supported in foxtail millet	(Rodriguez et al. 2010)

Root system architecture, a defining adaptive trait of millets, is also under strong miRNA control. Auxin related miRNAs such as miR160, miR167, and miR393 regulate lateral root initiation and elongation by targeting auxin response factor (ARF) genes and auxin receptors. These miRNAs have been widely shown to control root development in Arabidopsis and cereals (Iglesias et al. 2014; Liu et al. 2007) and are consistently identified in millet miRNA datasets under developmental and stress conditions (Jagadeesh Selvam et al. 2015; Palakolanu et al. 2022). In foxtail millet, SimiR396d, a member of the miR396 family, was experimentally shown to regulate root length and overall plant growth by targeting SiGRF1, directly linking miRNA mediated developmental regulation to drought adaptation (Zhang et al. 2023).

Leaf growth, canopy architecture, and biomass accumulation are regulated by miRNAs that control cell proliferation and expansion. miR396, which targets growth regulating factor (GRF) transcription factors, is a conserved regulator of leaf size and organ growth in grasses (Rodriguez et al. 2010) . Its presence and differential expression in millet species indicate a similar role in shaping leaf morphology and assimilate production (Chakraborty et al. 2020; Han et al. 2014). Likewise, miR319, which targets TCP (Teosinte branched1/cycloidea/proliferating cell factor) transcription factors, influences leaf shape, tillering, and photosynthetic capacity, traits that are critical for optimizing water-use efficiency in millet canopies (Palakolanu et al. 2022). Reproductive development in millets is tightly coordinated by the interaction between miR156 and miR172, where miR172 acts downstream of miR156 to promote floral transition by repressing APETALA2 like transcription factors. Conserved miR172 family members have been identified across millet genomes and are predicted to regulate flowering time and spikelet development, reinforcing their role in controlling phenological variable rainfall patterns (Chakraborty et al. 2020; Han et al. 2014). Collectively, miRNA-mediated developmental regulation provides millets with architectural and phenological flexibility, allowing them to balance vegetative growth and reproductive success under low-input and stress-prone environments. Recent genomic analyses in foxtail millet have further demonstrated the integration of miRNA prediction, transcription factor regulation, and haplotype variation in stress-responsive gene networks, providing additional resources for functional genomics and breeding applications (Rashid et al. 2026).

### miRNA-mediated regulation of nutritional and grain-quality traits.

The link between miRNAs and nutritional enhancement in millets, particularly grain micronutrient accumulation, grain quality, and bioavailability, remains under-investigated. Although millets possess an inherently superior nutritional profile rich in micronutrients such as iron and zinc, proteins, and bioactive compounds, direct evidence connecting specific miRNAs to improved grain nutritional traits is still limited. Most current knowledge is based on miRNA responses under nutrient deficiency conditions, predicted target relationships, or conserved evidence from other cereals rather than direct regulation of grain filling or micronutrient accumulation in millets. Notable targeted studies have begun to address this gap. In pearl millet, Mahendrakar et al. (2020) performed miRNA target analysis during an investigation of candidate genes for grain iron and zinc metabolism. They identified pgl-miR159 as a candidate associated with iron metabolism, representing one of the few millet specific attempts to link a miRNA with grain Fe-related pathways. However, this association should not be interpreted as functional validation of pgl-miR159-mediated grain Fe accumulation. Similarly, in foxtail millet, Li et al. (2024) conducted a genome wide miRNA analysis and identified candidate miRNAs predicted to be associated with starch biosynthesis genes and endosperm development. These findings provide useful targets for future grain quality research, but direct validation of the predicted miRNA target modules is still needed (Table 4).

**Table 4:** miRNA target interactions related to nutritional and grain-quality traits in millets with validation status

miRNA	Target gene(s)/pathway	Millet species	Validation	References
pgl-miR159	Iron metabolism-related genes	Pearl millet (*Pennisetum glaucum*)	Reported in millet through target/candidate analysis, but lacking direct functional validation	(Mahendrakar et al. 2020)
ptc-miR169i, fve-miR396e, mtr-miR162, PC-5p-221	Starch biosynthesis genes	Foxtail millet (*Setaria italica*)	Genome wide/predicted regulatory association; direct millet functional validation not yet available	(Li et al. 2024)
miR408	Phytocyanin/Copper homeostasis	Foxtail millet (*Setaria italica*) and pearl millet (*Pennisetum glaucum*)	Conserved module validated mainly in rice/Arabidopsis or other non-millet systems; millet specific validation remains limited	(Deng et al. 2022; Zhang et al. 2017)

## Integration of miRNA markers into marker-assisted breeding pipelines in millets

The development of diverse classes of molecular markers offers breeders and researchers greater flexibility in choosing suitable candidates for genetic breeding improvement (Tyagi et al. 2021; Yadav et al. 2014). A novel category of gene-based markers, known as miRNA markers, has been first described in Brassica species (Fu et al. 2013) and rice (Mondal and Ganie 2014). Sequence variations associated with miRNAs have been utilized to develop diverse classes of molecular markers, including SSRs, SNPs, AFLPs, SRAPs, and miRNA-based markers derived from conserved sequences (Fu et al. 2013; Hemasai et al. 2024). Although miRNA derived markers have been more extensively investigated in animal systems, their distinct advantages, such as high reproducibility and appreciable polymorphism, have attracted growing attention from plant researchers seeking to exploit their potential in crop improvement (Tyagi et al. 2021). Nevertheless, their direct application in plant breeding remains limited, primarily because of the relatively small number of known miRNAs, their short sequence length, and the generally low levels of polymorphism observed at these loci. Even so, miRNA-based markers are unique in the way they capture regulatory variation rather than merely neutral or structural differences, linking them directly to traits such as stress tolerance, developmental plasticity, and yield stability. This feature is particularly relevant for millets, where breeding for drought adaptation, nutrient-use efficiency, grain yield, and fertility is of paramount importance.

Recent advances in high-throughput sequencing have enabled the genome wide discovery of conserved and novel miRNAs in millets, along with their expression profiling across tissues and association with stress responses (Deng et al. 2022). These findings open opportunities to integrate miRNA polymorphisms and expression patterns into molecular breeding frameworks, offering breeders new tools to accelerate crop improvement. In parallel, advances in functional validation approaches such as degradome sequencing, target mimicry, STTM (Short Tandem Target Mimic), and CRISPR/Cas editing provide effective means to confirm and manipulate regulatory interactions (Djami-Tchatchou et al. 2017; Zhou et al. 2022). Finally, incorporating validated miRNA trait associations into marker-assisted and genomic selection models (Hemasai et al. 2024) represents a practical route to accelerate the development of stress-resilient and nutritionally enriched millet cultivars. In millets, several studies have highlighted their potential for integration into breeding pipelines. For instance, Yadav et al. (2014) pioneered the development of miRNA based genetic markers in foxtail millet by designing 66 primer pairs with robust amplification from conserved pre-miRNA sequences across foxtail millet cultivars and exhibited high cross-genera transferability, underscoring their promise as functional genotyping tools for both millet and related grass species. Likewise, Palakolanu et al. (2022) profiled root miRNAs in pearl millet under high vapor pressure deficit and found a greater diversity in the drought-tolerant genotype than in the sensitive one. Differentially expressed families such as miR165, miR168, miR170, miR319, miR167, miR172, miR396, and miR399 were associated with root related drought responses based on expression profiling and predicted targets, highlighting their value as candidate regulatory markers that require further validation before breeding deployment.

Such an integrated framework not only accelerates the development of stress-resilient and high yielding millet cultivars but also provides opportunities for nutritional biofortification. In cereals like rice, several studies have demonstrated that miRNAs regulate Fe/Zn transporter genes and thereby influence grain micronutrient accumulation. Paul et al. (2016) reported that high-iron transgenic rice lines exhibit the down regulation of root-specific miRNAs (*miR11, miR26, miR30*, and *miR31*), which in turn leads to the up regulation of key transporters such as *OsYSL15, OsFRO2,* and *OsIRT1*. The enhanced expression of these genes facilitates efficient uptake and translocation of iron and zinc, ultimately resulting in higher grain mineral content. This clear link between miRNA activity and transporter regulation provides a mechanistic framework that can be extended to millets, where identifying orthologous transporter miRNA modules and manipulating their expression could be a promising strategy for nutritional biofortification. Recent genome wide studies in pearl millet have uncovered orthologs of major transporter families, viz., Zinc-regulated transporter/Iron-regulated transporter like protein (ZIP), Natural Resistance Associated Macrophage Proteins (NRAMP), Yellow Stripe Like (YSL) transporters, Nicotianamine Synthase (NAS), and ferritin through transcriptomic and QTL-based approaches (Mahendrakar et al. 2020). These identified transporter orthologs provide a useful starting point for discovering candidate millet-specific miRNA transporter modules, but their use in biofortification will require direct validation of miRNA targeting, transporter regulation, grain mineral accumulation, and trait stability across genetic backgrounds and environments.

## Role of miRNAs in plant growth and stress response

In plants, miRNAs play essential roles for developmental processes such as organ differentiation, root and shoot architecture, leaf morphogenesis, flowering time, and seed development (Chen 2009). They fine tune hormonal signaling networks involving auxin, cytokinin, and gibberellin, thereby coordinating growth and morphogenesis under varying environmental conditions (Li and Zhang 2016). Under abiotic and biotic stress, miRNAs act as molecular switches that modulate adaptive responses. For example, miRNAs are key regulators under oxidative, sulfate, and phosphate starvation stresses in plants (Sunkar et al. 2006; Bari et al. 2006). Similarly, miRNAs also modulate tolerance to drought, salinity, and pathogen attack by targeting transcription factors such as MYB, NAC, and GRF families (Zhang 2015). The following section covers the key roles of miRNAs in stress management and nutrient transport and draws insights for future research on miRNA research in millets.

### miRNAs identified for stress responses in model plant (Arabidopsis)

The model plant *Arabidopsis thaliana* has been extensively used to understand the miRNAs involved in the stress regulation. Numerous studies on the regulation of miRNAs under biotic and abiotic stress conditions have been published, and researchers are now investigating these miRNAs to ascertain their exact functions and potential use in agriculture.

### Abiotic stress

The most significant abiotic stressor influencing plant development and production is extreme or extended heat stress. Heat regulated miRNAs may enhance a plant's resistance to heat stress by forming extensive regulatory networks with their target genes. Numerous metabolic activities are regulated by these networks, including miRNA biogenesis, protein refolding, antioxidant defense, photosynthetic system maintenance, reproductive tissue protection, and flowering time control (Ding et al. 2021). Multiple miRNAs were found in Arabidopsis as responsive to heat stress. miR156 was identified as significant in adjusting to recurrent heat stress, since it is functionally essential for heat stress memory. Subsequent to heat stress, miR156 exhibited significant upregulation, targeting and post transcriptionally downregulating SPL (*SQUAMOSA promoter binding like*) transcription factor genes, which are essential master regulators of developmental transitions and pivotal for heat stress memory (Stief et al. 2014). MiRNA mediated changes in plant development were noted under heat stress by miR160 (Lin et al. 2018) and miR164 (Tsai et al. 2023). Heat stress induced overexpression of miR160 that targets and represses the expression of *auxin response factors,* ARF10, ARF16, and ARF17, regulating seed germination, hypocotyl growth, and rachis growth. The expression levels of these miR160 targets altered *heat shock protein* (*HSP*) expression and further regulated the heat stress responses in Arabidopsis (Lin et al. 2018). Similarly, overexpression of miR164 and repression of its targets were observed under long term heat stress, conferring enhanced thermotolerance. Under long-term heat stress, miR164 is upregulated, which suppresses its target genes and triggers a cascade of physiological changes. The result includes shifts in the chlorophyll a/b ratio, altered HSP (heat shock protein) expression, increased H₂O₂-driven oxidative damage, and disturbances in plant development. Together, these effects determine the plant’s overall thermotolerance (Tsai et al. 2023). Heat stress rapidly induced miR398 and heat stress transcription factors (HSF genes), *HSFA1b* and *HSFA7b,* were responsible for heat induction of miR398. The up regulated *miR398* conferred thermotolerance through down regulation of its target genes, *CSD1*, *CSD2* (copper/zinc superoxide dismutase) and *CCS* (Copper Chaperone for Superoxide Dismutase) (Guan et al. 2013).

Cold temperatures, comprising both chilling (less than 20 °C) and freezing (less than 0 °C) injury, are one of the main abiotic stresses that affect plant growth and yield (Thakur et al. 2010). Cold stress activates a large number of responsive genes that encode transcription factors (Lee et al. 2005). Potential miRNAs such as miR394, miR397, and miR169 were known to be involved in cold stress response. miR169 showed the potential role of the miR169/NF-YA module in the cold stress response mediated by Aux/IAA14 (Aslam et al. 2020). Overexpression of miR394 and miR397 improved tolerance to cold stress, where upregulated *miR397a* affected the expression of cold regulated *CBF* (CRT/DRE binding factor) genes and downstream cold-regulated (*COR*) genes (Dong and Pei 2014). Similarly, miR394-regulated cold tolerance was involved in the CBF dependent cold responsive pathway (Song et al. 2013). Transcription factors known as C repeat binding factors (CBFs)/dehydration responsive element binding factors 1 (DREB1s) are of particular interest in cold stress response pathways because they can bind to the C repeat/dehydration responsive element (CRT/DRE) and activate a large number of downstream genes (Medina et al. 2011; Song et al. 2013).

In most regions of the world, drought is a common and recurrent climatic condition that significantly reduces crop yield (Ferdous et al. 2015). Plants’ primary survival responses against drought stress are early flowering and osmotic potential adjustment. Sugar, with its role in osmotic adjustment and the transition from vegetative to reproductive growth, is an ideal signal that can connect both strategies (Han et al. 2015; Seo et al. 2011). Both drought responses might be influenced by ABA, another significant drought induced component. ABA helps plants become more drought tolerant (Lu et al. 2009; Muhammad Aslam et al. 2022). Han et al. reported *GIGANTEA *(*GI*) dependent up-regulation of *miRNA172E* by drought stress that down regulated *WRKY44* (involved in sugar signaling), thus regulating drought escape and drought tolerance by affecting sugar signaling in Arabidopsis (Han et al. 2015). A study in transgenic *Arabidopsis thaliana* revealed that overexpression of gma-MIR394a in Arabidopsis reduced the transcript of an F-box gene (At1g27340) having a miR394 complementary target site, resulting in plants with lower leaf water loss and improved drought tolerance (Ni et al. 2012). It was reported by Yang et al. that miR165/166 mediated *HD-ZIPIII* regulation (Class III *Homeodomain-Leucine Zipper* (*HD-ZIP III*) family genes) confers drought tolerance through ABA signaling (Yang et al. 2019).

Studies on the regulation of the salt stress response pathway in Arabidopsis revealed that down-regulation of auxin signaling through RACK1A-induced miR393 biogenesis potentially regulates the Arabidopsis acclimation to salinity (Denver and Ullah 2019), and salinity-induced expression of miR393 negatively regulates TIR1 and AFB2 receptors related to auxin signaling and specific redox-associated components (Iglesias et al. 2014). It was reported that under salt stress conditions, miRNA417 functions as a negative regulator of seed germination in Arabidopsis (Table 3) (Jung and Kang 2007). For combined abiotic stresses, different miRNAs such as *osa*-*MIR393* for salinity and alkaline stress (Gao et al. 2011), miR394 for salt and drought stress (Song et al. 2013), miR408 for salinity, cold, and oxidative stress (Ma et al. 2015), miR172c for water deficit and salt stress (Li et al. 2016), miR399f for salt stress, ABA signaling, and drought stress (Baek et al. 2016), and miR156, miR319a/b, miR319b.2, and miR400 for a wide range of abiotic stresses (Barciszewska-Pacak et al. 2015; Cui et al. 2014) were reported in Arabidopsis involved in the abiotic stress regulation pathway (Table 5).

**Table 5:** miRNA responsive to abiotic stresses in the model plant Arabidopsis. Details on the type of stress, name of the miRNA, and roles are listed with references

S.No	Stress	miRNA	Role/strategy	Reference
1	Heat stress	miR164	Overexpression of miR164 conferred enhanced thermotolerance	(Tsai et al. 2023)
		miR160	Heat stress increased the expression of miR160	(Lin et al. 2018)
		*miR398*	Down-regulation of *CSD* genes and *CCS*	(Guan et al. 2013)
2	Cold stress	miR169	Aux/IAA14-mediated cold stress response	(Aslam et al. 2020)
		miR394	Overexpression of miR394a promotes transcription of major cold responsive genes	(Song et al. 2016)
		*miR397*	Overexpression of miR397a causes increased CBF transcript levels leading to induction of cold responsive COR genes	(Dong and Pei 2014)
3	Drought stress	miR165/166	miR165/166- mediated *HD-ZIP IIIs* regulation	(Yang et al. 2019)
		*miRNA172*	Suppression of WRKY44 by GIGANTEA-miR172	(Han et al. 2015)
		miR394a	Overexpression of *gma-MIR394a* reduces the transcript of an F-box gene (*At1g27340*)	(Ni et al. 2012)
		miR169	Downregulation of miR169 contributes to the transcript accumulation of *NFYA5*	(Li et al. 2008)
4	Salt stress	miR393s	Regulates scaffold protein RACK1A mediated ABA signaling pathways	(Denver and Ullah 2019)
		miRNA417	Negative regulator of seed germination under salt stress conditions	(Jung and Kang 2007)
	Salinity	miR393	miR393 regulation of the TIR1 and AFB2 receptors	(Iglesias et al. 2014)
	Salt stress, ABA signaling and drought stress	miR399f	Regulates expression of ABF3 and CSP41b genes	(Baek et al. 2016)
	Water deficit and salt stress	miR172c	Silencing of *Glyma01g39520* mRNA	(Li et al. 2016)
	Salinity, cold and oxidative stress	*miR408*	Elevated *miR408* expression enhances cellular defence against oxidative stress	(Ma et al. 2015)
	Salt and drought stresses	miR394	Silencing of *LCR* mRNA by miR394	(Song et al. 2013)
5	Salinity and alkaline stress	*osa*-*MIR393*	Regulates salt and alkali stress responsive genes	(Gao et al. 2011)
6	Abiotic stresses	miR319a/b, miR319b.2, and miR400	Gene expression regulation in plant response to a wide array of abiotic stresses	(Barciszewska-Pacak et al. 2015)
		*miR156*	During stress, *miR156-SPL9-DFR* pathway coordinates and *SOC1* expression is reduced to maintain the plant in a relatively long juvenile state	(Cui et al. 2014; Stief et al. 2014)

### Biotic stress

Biotic stresses cause significant agronomic losses each year, and plant miRNAs have emerged as key regulators of immunity and defense signaling pathways. One major defense regulator, jasmonate (JA), plays a pivotal role in insect resistance. Mao et al. (2017) demonstrated that JA responses decline in Arabidopsis, and this process is modulated by the miR156 SPL9 regulatory module. Elevated accumulation of miR156 during the juvenile stage represses SPL9, which normally stabilizes the JASMONATE-ZIM DOMAIN protein 3 (a repressor of JA signaling). Consequently, repression of SPL9 leads to activation of the JA pathway, enhancing resistance to cotton bollworm infestation.

Beyond insect defense, several miRNAs are implicated in plant pathogen interactions. For instance, miR773 functions as a negative regulator of pathogen-associated molecular pattern (PAMP) triggered immunity in Arabidopsis. Salvador-Guirao et al. (2018) showed that suppression of miR773 enhances resistance by relieving this repression. Similarly, downregulation of miR396 triggers hyperactivation of defense responses, including hydrogen peroxide accumulation and callose deposition, thereby strengthening resistance against fungal pathogens (Soto-Suárez et al. 2017). In bacterial defense, miR472 was found to target the miR472–RDR6 pathway, repressing a subset of *CC-NB-LRR *(*CNL*) mRNAs at the post-transcriptional level (Table 6). This regulation modulates both PAMP- and effector triggered immunity (Boccara et al. 2015). Interestingly, miRNA mediated regulation also integrates responses to simultaneous stresses. Under combined drought and bacterial infection, miR164c was shown to regulate expression of the *AtP5CS1* gene in the proline biosynthesis pathway, thereby linking abiotic and biotic stress responses (Gupta et al. 2020). Collectively, these findings highlight the multifaceted role of miRNAs as central modulators of plant immunity, acting through fine-tuned regulatory networks that balance growth, defense, and adaptation.

**Table 6:** miRNA responsive to biotic stresses in the model plant Arabidopsis

S.No	Stress	miRNA	Role/strategy	Reference
1	Fungal pathogens	miR773	miR773 mediates pathogen-associated molecular pattern-triggered immunity	(Salvador-Guirao et al. 2018)
		miR396	Reduced miR396 activity contributes to superactivation of defense responses	(Soto-Suárez et al. 2017)
2	Combined stress of drought and bacterial infection	*miR164c*	Regulates expression of *AtP5CS1* gene	(Gupta et al. 2020)
	Bacterial disease	*miR472*	*miR472-RDR6* Silencing	(Boccara et al. 2015)

## Role of miRNA in regulation of nutrient deficiency in plants

Plants often encounter environments with suboptimal mineral nutrient availability, requiring them to continuously monitor and respond to fluctuations in ion concentrations within their surroundings. To maintain proper metabolic function, growth, reproduction, and defense, nutrient uptake and internal demand must be precisely coordinated among different organs. Plants absorb mineral ions from the soil and transport them to various tissues through both long- and short-distance pathways (Wolswinkel 2024). The molecular mechanisms underlying ion uptake, translocation, and storage are governed by the differential expression of transporter proteins that adjust according to nutrient availability (Amtmann and Blatt 2009). miRNAs function as crucial regulators of nutrient stress responses, ensuring nutrient homeostasis by modulating the expression of genes involved in nutrient acquisition and distribution (Paul et al. 2015).

Nitrogen is a vital macronutrient essential for the growth and development of plants, required in substantial quantities within plant cells. It serves not only as a fundamental component of amino acids, nucleic acids, and chlorophyll but also plays a crucial regulatory role in carbon and amino acid metabolism as well as protein synthesis (Cai et al. 2012). Plants absorb nitrogen from the soil primarily in the forms of ammonium and nitrate through specialized transport proteins located in root cells. This nutrient typically constitutes between 1 and 5% of the total dry mass of plants (Fukuda et al. 2020). miRNAs are key regulators of nitrogen responsive pathways. For instance, miR158 targets pentatricopeptide repeat containing proteins and fucosyl transferases, modulating symbiotic interactions under N limitation (Liang et al. 2012). Under N deficiency, miR160 is upregulated while miR169 is downregulated in Arabidopsis (Nguyen et al. 2015). Increased miR160 suppresses auxin response factors (ARF16 and ARF17), thereby influencing auxin mediated growth regulation (Liu et al. 2007). Conversely, the downregulation of miR169 induces overexpression of NF-Y genes, which enhance nitrate transporter activity (AtNRT1.1 and AtNRT2.1), improving nitrogen uptake efficiency (Zhao et al. 2012).

Multiple miRNAs, including miR160, miR169, miR171, miR395, miR397, miR398, miR399, miR408, and miR827, have been implicated in nitrogen deficiency responses in Arabidopsis and maize (Nguyen et al. 2015). In maize, N limitation also alters the expression of conserved (e.g., miR169, miR395, miR528, miR827) and novel miRNAs (e.g., miRC1, miR169w, miRC19, miRC37, miR171p/q) (Zhao et al. 2012). In wheat, TaMIR444a has been identified as a nitrogen deprivation responsive miRNA contributing to adaptation under low N conditions (Gao et al. 2016). Similarly, in sorghum, miRNAs such as miR169s and miR399s respond to both nitrogen and phosphorus starvation and also exhibit regulation under potassium deficiency. Novel miRNAs, including miR5565c, miR5564, and miR1432, have been identified, suggesting previously unrecognized roles in multi-nutrient stress responses (Zhu et al. 2021).

Phosphorus (P) is an essential component of nucleic acids, lipids, ATP, and phosphorylated sugars, playing vital roles in energy dependent processes such as photooxidative and substrate-level phosphorylation. Inorganic phosphate (Pi) also regulates major metabolic pathways, enzyme activities, and signaling cascades (Grennan 2008). In Arabidopsis, miR827 regulates phosphorus homeostasis by repressing the *NLA* (*nitrogen limitation adaptation*) gene, while *NLA* mutations disrupt the expression of P transport related genes (*PHF1* and *PHT1*) and cause excessive P accumulation under P deficiency, indicating its nutrient-dependent regulatory role (Lin et al. 2013). Similarly, miR399 functions as a shoot-to-root signal that maintains Pi homeostasis via phloem transport and is capable of long-distance translocation under low P conditions (Huen et al. 2017; Rouached et al. 2010). Numan et al. (2022) identified 161 known and 348 novel miRNAs in *tef*, with predicted targets involved in macronutrient uptake and transport, particularly calcium and phosphate. These findings highlight the pivotal role of miRNAs in regulating ion homeostasis and adaptive responses during nutrient stress.

Beyond its roles in photosynthesis, osmoregulation, and enzyme activation, potassium (K) plays a vital role in numerous physiological and metabolic processes in plants. It is essential for maintaining cell turgor, promoting cellular expansion, regulating the electric potential across cell membranes, and maintaining intracellular pH homeostasis. Moreover, K influences both transcriptional and post transcriptional regulation, thereby modulating several growth-related pathways. In wheat, functional characterization of tae-miR408, which is markedly downregulated under K deficiency, revealed its crucial role in mediating tolerance to K limitation. Transgenic tobacco plants overexpressing *tae-miR408* exhibited significantly enhanced K uptake, biomass accumulation, photosynthetic efficiency, and reactive oxygen species (ROS) scavenging compared to wild-type plants under K-deficient conditions (Zhao et al. 2020).

## miRNAs in grain development, yield, yield-associated parameters, and quality traits

Yield remains the most critical yet complex trait targeted in crop improvement. Although significant progress has been made in dissecting its genetic basis, the architecture and underlying determinants of yield remain only partially understood. Fundamentally, component traits like tiller number, seed weight, and seed number directly influence crop yield. Emerging evidence indicates that miRNAs also play a vital role in modulating these traits through regulation of genes involved in branching, biomass accumulation, and overall plant architecture.

Early work by Schwab et al. (2005) demonstrated that overexpression of *miR156* in Arabidopsis significantly increased branching, resulting in more than a 300% enhancement of biomass. This effect is mediated through miR156 targeting members of the SQUAMOSA promoter binding like (SPL) transcription factor family. Similar outcomes were reported in rice and tomato, where elevated miR156 expression promoted biomass accumulation (Xie et al. 2012). More recently, Song et al. (2018) showed that overexpression of miR408 in Arabidopsis altered leaf area, petiole length, and silique length, ultimately increasing biomass and seed quality. These effects were attributed to enhanced myosin gene expression and elevated gibberellic acid levels, which together facilitated greater cell expansion.

The first direct evidence of yield improvement through miRNA technology came from *osa-miR397* overexpression, which increased grain size and yield by up to 25% in rice (Zhang et al. 2013a, b). Research has shown that miRNAs play a pivotal role in rice yield regulation; *OsSPL14*, targeted by *osa-miR156*, enhances panicle branching and yield when overexpressed (Miura et al. 2010; Jiao et al. 2010). Other instances include overexpression of *MIR444a* regulating tillering via the OsMADS57 and D14 downregulation network (Guo et al. 2013), suppression of *OsGA20ox2* improving grain number and weight (Qiao et al. 2007), and yield increases mediated by *osa-miR408* and *osa-miR1873* (Zhang et al. 2017; Zhou et al. 2020). Collectively, these findings highlight miRNAs as powerful tools for yield improvement. In addition, yield related traits including biomass, harvest index, plant architecture, and adaptation, as well as resistance to biotic and abiotic stresses, indirectly influence yield, either by modifying the yield components or through other pathways (Shi et al. 2009). Advances in functional genomics have revealed that miRNAs play crucial roles in regulating these traits. By modulating the expression of genes associated with plant architecture and yield related pathways, miRNAs provide opportunities for targeted yield improvement. Furthermore, grain yield and quality represent pleiotropic traits shaped by cooperative genetic factors, with miRNAs and their regulatory networks serving as integral components of this complex genetic framework. miRNA reported to be involved in starch biosynthesis has been identified in foxtail millet (Li et al. 2024) and also upregulated methionine and folate metabolism under exogenous application of salicylic acid in foxtail millet (Hou et al. 2022).

## Comparative insights into miRNA research in millets and major cereals

Millets possess unique miRNA-mediated regulatory networks that contribute to adaptation under drought and other abiotic stresses. Although stress-responsive miRNAs are also widely reported in major cereals, differences in miRNA repertoires and regulatory strategies may underlie the superior adaptation of many millet species to arid and marginal environments (Palakolanu et al. 2022). Research on millet miRNAs has expanded considerably in recent years; however, it remains less comprehensive than the extensive functional characterization and trait-level applications reported in rice, wheat, maize, and barley (Singroha et al. 2021). Current studies in millets largely focus on the identification of conserved and novel miRNAs and their stress-responsive expression patterns using small RNA sequencing, degradome analysis, and qRT-PCR. For example, Chakraborty et al. (2020) summarized the structural and functional characteristics of miRNAs across five strategic millet species and highlighted their importance in drought adaptation. More recently, Rani et al. (2025) identified the Eco-miR169–EcNF-YA13 regulatory module as a key determinant of dehydration and salinity tolerance in finger millet.

In contrast, cereals benefit from extensive functional investigations employing overexpression, knockdown, short tandem target mimic, and CRISPR-based approaches, which have established the roles of miR159, miR164, and miR398 in regulating flowering, yield-related traits, and stress responses (Lian et al. 2024; Lin et al. 2021). In addition, integrated multi-omics analyses combining small RNA sequencing, degradome profiling, transcriptomics, proteomics, and metabolomics have facilitated the reconstruction of complex miRNA-regulated networks in cereals (Tong et al. 2025). Comparable integrative studies remain relatively limited in millets, where most investigations are still centered on small RNA and degradome datasets (Li et al. 2024). Expanding multi-omics approaches in millet research will therefore be important for linking miRNA regulation with physiological and metabolic processes and for achieving a systems-level understanding of stress adaptation.

## Future prospects for miRNA research in millets

Despite significant advances in the identification and characterization of stress-responsive miRNAs in millets, several knowledge gaps remain. Future progress will depend on the integration of high-throughput small RNA sequencing, degradome analysis, transcriptomics, proteomics, and metabolomics to achieve a systems-level understanding of miRNA-mediated regulatory networks. Large-scale profiling across diverse millet species, tissues, developmental stages, and environmental conditions will facilitate the discovery of novel miRNAs and their targets, thereby expanding current genomic resources (Bonnet et al. 2004; Chandra et al. 2024). Because many miRNA targets encode transcription factors and other key regulatory proteins, comprehensive studies integrating miRNA and mRNA expression data will be essential for deciphering complex regulatory pathways governing growth, development, and stress adaptation (Samad et al. 2017; Zhang 2015). Furthermore, computational predictions should be complemented by rigorous experimental validation using degradome sequencing, RLM-RACE, PPM-RACE, qRT-PCR, and reporter-based assays to confirm miRNA–target interactions (Wang and Fang 2015).

Advances in reverse genetics and genome editing technologies, including overexpression, knockout, and CRISPR/Cas-mediated manipulation of miRNAs and their target genes, provide powerful tools for elucidating miRNA function in millets. Insights gained from well-characterized cereal systems, such as the *OsmiR530* and *OsPIL15–OsmiR530* regulatory modules in rice, offer valuable frameworks for identifying conserved and species-specific regulatory mechanisms in millet crops (Mandal et al. 2021; Sun et al. 2020). Such efforts will accelerate the development of predictive regulatory models and deepen our understanding of miRNA-controlled traits in millets.

### Building comprehensive miRNA atlases in millets

**Fig. 2:**
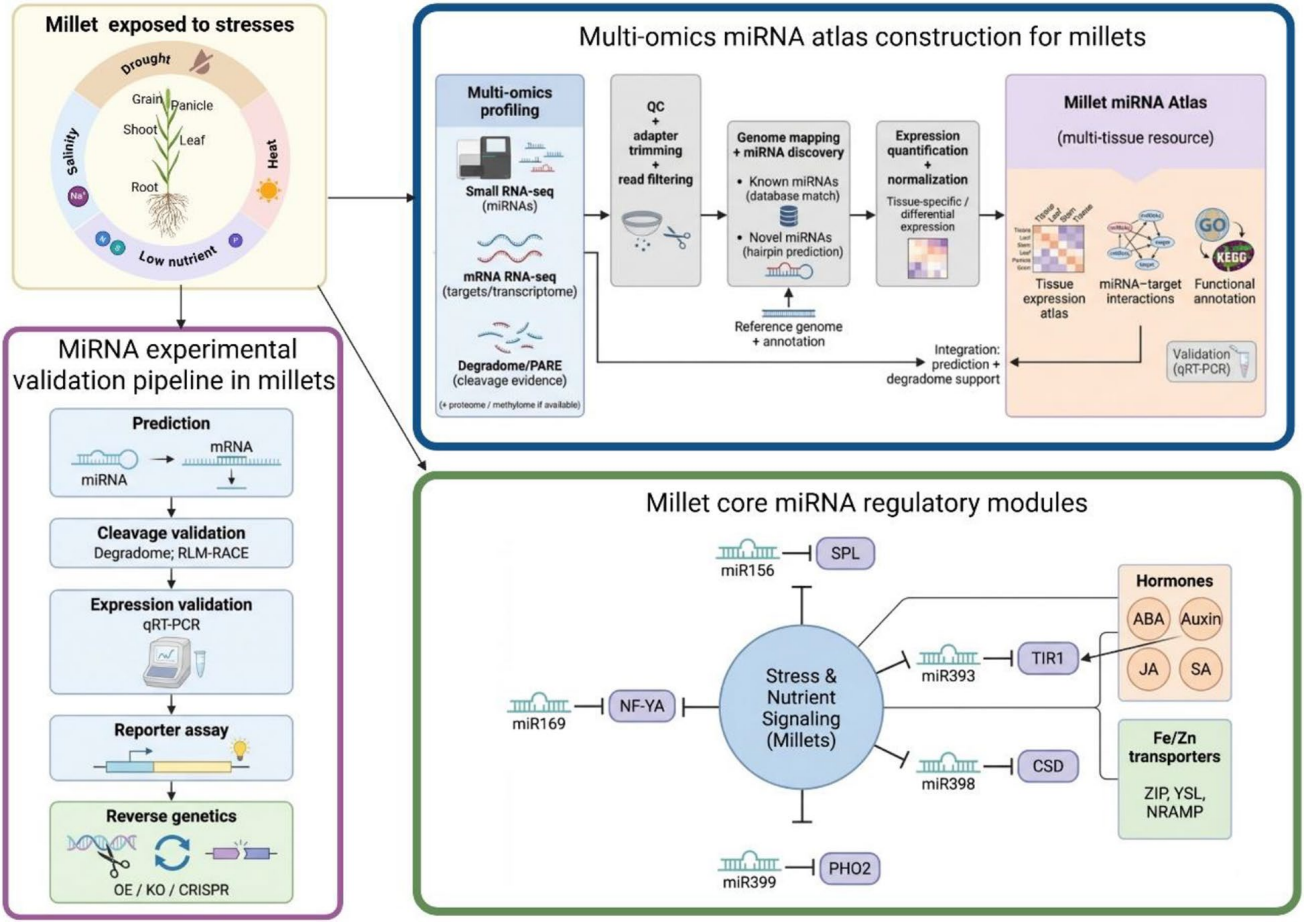
Multi-omics framework and functional modules of miRNA regulation in millets. Millets exposed to drought, salinity, heat, and nutrient stress are profiled using integrated small RNA-seq, transcriptome, and degradome analyses to construct a comprehensive multi-tissue miRNA atlas. The workflow includes quality control, genome mapping, novel miRNA discovery, expression normalization, and miRNA-target interaction annotation with degradome support. Experimental validation pipelines incorporate cleavage assays, qRT-PCR, reporter analyses, and reverse genetics. Core regulatory modules, miR156–SPL, miR169–NF-YA, miR393–TIR1, miR398–CSD, and miR399–PHO2, coordinate stress, hormonal, and micronutrient signaling, linking molecular regulation to adaptive growth and nutrient homeostasis can be explored in millets

High-resolution, multi-factorial miRNA atlases are urgently needed in millets. Future work should deploy sRNA seq across combined drought, heat, salinity, cold, and macro/micro nutrient (N, P, K, Fe, Zn) stresses (Fig. 2). Sampling can be done in root, shoot, panicle, and grain filling tissues in contrasting elite vs. landrace genotypes to capture hidden regulatory diversity (Chakraborty et al. 2020; Wang et al. 2016). Systematic discovery of millet specific and lineage restricted miRNAs, expanding on current in silico and NGS resources, will help disseminate conserved stress modules (ROS detoxification, osmoprotection) from unique C4 and deep root-associated networks that underline climate resilience. Systematic comparisons with rice, wheat, and maize miRNAomes under matched conditions will further delineate millet specific regulatory modules from grass wide stress hubs (Shinde et al. 2020). Time series sampling during stress onset, progression, recovery, and reproductive stages is essential to identify early “sentinel” miRNAs predicting drought and heat failures and to characterize grain specific miRNAs governing the remobilization and loading of Fe, Zn, and other micronutrients into developing grains (Paul et al. 2015). Ultimately, integrating these temporal spatial miRNA datasets with transcriptomic, proteomic, and ionomic frameworks will enable construction of causal miRNA target trait networks, nominating candidates for genome editing and miRNA guided breeding to enhance climate resilience and nutritional quality in millets (Palakolanu et al. 2022).

### Climate-smart breeding driven by miRNA markers in millets

DNA markers play a central role in molecular breeding, and a wide range of marker systems have been developed to accelerate crop improvement programs. Among them, miRNA based molecular markers represent a distinct class of functional markers that have been extensively applied in animal systems but reported in only a few plant species, such as rice (Zheng and Qu 2015) and Brassica (Fu et al. 2013). Owing to their high efficacy, stability, and superior cross-genera transferability, miRNA-based markers provide a promising opportunity for millet molecular breeding. Importantly, integrating miRNA-based markers into marker-assisted breeding pipelines can facilitate the precise identification and manipulation of key genetic targets associated with stress tolerance, yield stability, and nutritional enhancement. In the broader context of climate change and food security, this approach offers a powerful strategy to accelerate the development of climate resilient and nutritionally enriched millet cultivars, thereby strengthening the role of millets as a cornerstone of sustainable food systems.

Decoding stress-responsive miRNA target networks in millets requires systems-level integration of sRNA, degradome, transcriptome, and proteome datasets to reconstruct regulatory modules controlling climate resilience. Core hubs such as the miR156 SPL pathway, which modulates heat and drought plasticity and developmental transitions, have been widely reported across cereals (Cui et al. 2014). Similarly, the miR169–NF-YA module, a central regulator of drought sensitivity and guard cell function, is highly conserved and likely to fine-tune water-deficit responses in millets (Li et al. 2008). The miR393–TIR1 auxin receptor circuit regulates root system architecture under stress and is an important candidate for understanding root foraging strategies in low moisture soils (Iglesias et al. 2014). Oxidative stress regulation through miR398 CSD homeostasis represents an additional layer of protection against drought and heat stress (Sunkar et al. 2006), while miR399–PHO2 remains the central module governing phosphate starvation responses and nutrient remobilization (Bari et al. 2006).

Integrating ionomic and metabolomic datasets with these miRNA modules is critical for linking miRNA signatures to antioxidant metabolites, osmolytes, and mineral accumulation in millets. miRNAs regulating Fe/Zn transporters such as *ZIP, YSL,* and *NRAMP* families, as well as genes controlling phytic acid biosynthesis, are promising targets for improving nutritional quality in millets (Paul et al. 2016). Unraveling hormonal miRNA cross-talk will further clarify stress-regulatory circuits in millets such as ABA induced miRNAs that modulate drought response, auxin-linked miRNAs shaping roots under low moisture, and ethylene/SA/jasmonate responsive miRNAs driving heat tolerance and defense (Curaba et al. 2014). Together, these multi-omics and hormone miRNA integration strategies will enable construction of causal miRNA trait networks for breeding climate resilient, nutrient rich millets.

### Nutritional enrichment via miRNA engineering in millets

Nutritional enrichment in millets via miRNA engineering will depend on elucidating miRNA-controlled modules governing micronutrient uptake, partitioning, and grain loading. First, systematic identification of miRNAs targeting nutrient transporters (*ZIP, YSL, IRT1/2, NRAMP, NAS*) and seed storage components in pearl millet, finger millet, foxtail millet, and other millets is needed, integrating small RNA, degradome, and ionomic datasets to connect specific miRNA shifts with grain nutrient phenotypes (Mahendrakar et al. 2020). Parallel efforts should focus on miRNAs modulating phytic acid biosynthesis and its regulators so that editing or silencing of key miRNA nodes can simultaneously enhance mineral density and bioavailability, complementing ongoing biofortification pipelines in pearl millet (Vinoth and Ravindhran 2017). Second, grain-stage-specific miRNA atlases in developing panicles and endosperm should be constructed to pinpoint small RNAs that control nutrient loading, starch architecture, protein composition, and essential amino acid biosynthesis, enabling targeted manipulation of a few grain miRNAs to stack nutritional and processing traits in climate resilient backgrounds. Finally, the miRNA hormone nutrient triad must be dissected in millet roots by linking P/N foraging circuits governed by miR399, miR395, and miR827 to auxin and ABA signaling and to whole-plant mineral profiles under drought and low-input conditions (Fasani et al. 2021; Islam et al. 2022). Such integrated frameworks will prioritize millet miRNA targets for genome editing or miRNA assisted breeding to jointly enhance nutritional quality and climate resilience.

### Harnessing millet diversity and wild relatives for miRNA research

Exploiting the enormous diversity of landraces and wild relatives of millets offers a direct path to discovering novel miRNA alleles associated with climate resilience and nutritional quality. Genome and pan-genome resources in *Setaria viridis* and foxtail millet already reveal domestication sweeps and adaptive loci that can be coupled with sRNA and degradome maps to pinpoint stress-responsive miRNA target modules (Hu et al. 2018; Mamidi et al. 2020). Landrace panels and wild relatives across *Setaria, Eleusine, Panicum,* and *Cenchrus* capture allelic richness for drought, heat, low N/P and micronutrient efficiency, and can be systematically mined for miRNA variants linked to these traits using integrated omics and high throughput phenotyping pipelines (Aggarwal et al. 2022; Huang et al. 2016). Comparative genomics between orphan crops and their wild relatives further illustrates how domestication and modern breeding can erode resilience-associated diversity, while retaining or reshaping nutritional attributes (Maharajan et al. 2024; Ye and Fan 2021). Future work in millets should therefore combine dense resequencing, miRNA profiling, and GWAS/QTL mapping across landrace wild introgression populations to identify “lost” or underutilized miRNA alleles, and design genomic selection schemes that introgress beneficial miRNA modules into high yielding, farmer preferred cultivars without sacrificing grain quality or adaptation. Such evolutionary guided approaches will help design next-generation millet varieties that combine ancestral robustness with modern nutritional and productivity goals.

### Creating predictive models and digital miRNA twins in millets

**Fig. 3:**
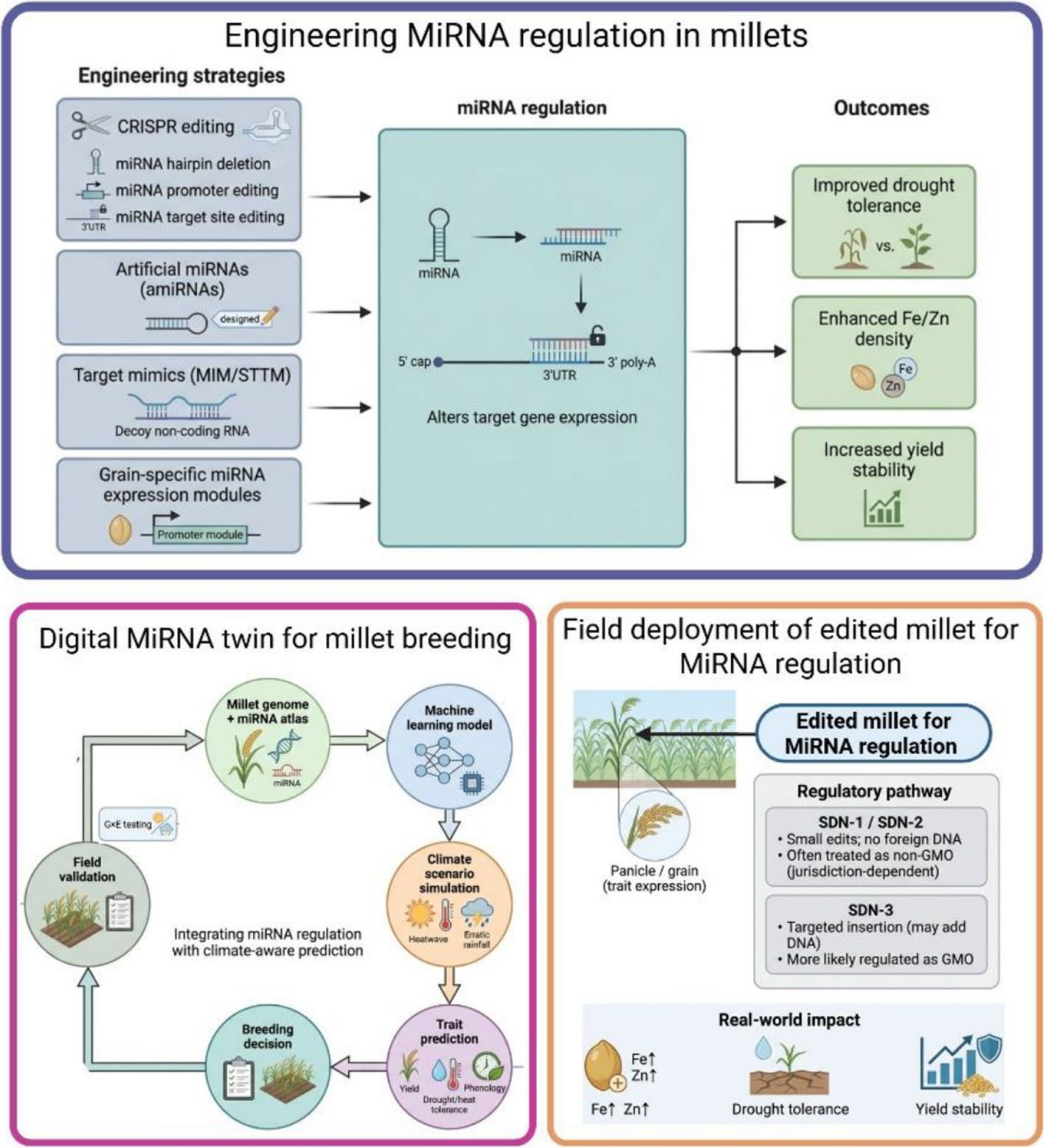
Engineering, digital modeling, and deployment of miRNA regulation in millets. Diverse engineering strategies, including CRISPR-based editing of miRNA loci, promoters, or target sites, artificial miRNAs (amiRNAs), target mimics (MIM/STTM), and grain-specific expression modules, enable precise modulation of miRNA activity and target gene repression. These interventions reshape stress-responsive and nutrient-regulatory networks, leading to improved drought tolerance, enhanced Fe and Zn biofortification, and greater yield stability. Integration of millet genome resources and miRNA atlases with machine learning supports a “digital miRNA twin” framework for climate-aware trait prediction and breeding decisions. Edited lines are subsequently evaluated for regulatory classification (SDN-1/2 versus SDN-3) and validated under field conditions to ensure translational impact

Developing predictive models and digital miRNA twins represents the next frontier in millet improvement, enabling in silico forecasting of stress responses and nutrient outcomes (Fig. 3). Machine learning frameworks can be trained to predict stress responsive miRNAs using structural hairpin motifs, sequence conservation, evolutionary signatures, and promoter architecture. Deep learning approaches have already demonstrated strong capacity to classify plant miRNAs and infer functional modules from sequencing alone (Zou et al. 2019a, b). Applying these models to diverse millet genomes and landrace datasets will allow prioritisation of candidate miRNAs regulating drought, heat, and nutrient stress before field validation. A complementary direction is the integration of miRNA regulatory layers into crop simulation platforms. Current climate responsive crop models successfully simulate phenology, biomass, yield, and stress responses under variable rainfall and temperature regimes (James et al. 2017). Embedding miRNA target interactions that control root architecture, senescence, osmotic adjustment, and nutrient remobilisation into these mechanistic frameworks would enable “miRNA-aware” simulations capable of predicting how edited or introgressed miRNAs perform across drought cycles, heatwaves and nutrient poor soils in millets. Ultimately, digital miRNA twins, dynamic computational avatars of regulatory networks, could guide trait engineering, assist genomic selection pipelines, and reduce breeding cycles for climate resilient millets.

### Translational pathways with miRNAs for climate resilient and nutrient dense millet cultivars

Millet miRNA resources (e.g., foxtail millet) now enable trait targeted editing for stress resilience and nutrition. Multilocation trials should compare edited lines across arid, semi-arid, and favorable sites under managed drought/heat versus optimal management, quantifying grain Fe/Zn, grain quality, and yield stability with AMMI/GGE to capture Genotype × Environment (Gangashetty et al. 2021). Benchmarks should align with pearl millet Fe and Zn targets adopted in India (≥ 42 mg kg⁻^1^ Fe; ≥ 32 mg kg⁻^1^ Zn) while safeguarding competitive yield (Govindaraj et al. 2019). Programs must distinguish non transgenic edits for Site Directed Nucleases-1 and 2 from transgenic routes (SDN-3) and document the absence of exogenous DNA. India’s 2022 guidelines exempt SDN-1 and 2 end-products from GMO-style environmental risk assessment once vector sequences are segregated (Department of Biotechnology India 2022). Environmental safety should also consider the mobility of plant miRNAs within and between organisms and its implications for non-target effects, even if edits are cisgenic (Loreti & Perata 2022). Coupling miRNA engineering (for drought and heat tolerance and nutrient remobilization) with genomic selection and high-throughput phenomics can accelerate quality millet production under stress-prone and marginal environments. CRISPR/Cas knockout or editing of specific miRNA families offers a tractable route to validate targets controlling stress and quality traits prior to breeding deployment (Zhou et al. 2017). Prioritize ideotypes combining early vigor, stay green, and micronutrient dense grain while retaining farmer preferred processing or culinary traits to drive adoption.

Millets’ inherent drought/heat resilience and micronutrient richness make them ideal testbeds for miRNA-guided climate-smart breeding and management. An expanding foxtail millet miRNA regulatory map supports target discovery and circuit design, while pearl millet provides a high impact use case for resilient, Fe and Zn dense cultivars integrated with digital agronomy. Establishing open reference panels linking miRNA states, phenomics, and input responses will position millets as global models for genetic resilience, nutritional density, and smart agriculture.

### Role of CRISPR/Cas tools in millet miRNA research

CRISPR based genome editing has emerged as a transformative approach in plant functional genomics, providing unprecedented opportunities to dissect and manipulate the roles of non-coding RNAs, particularly miRNAs (Fig. 2). Beyond their genomic sequences, the secondary structures of miRNAs are critical for accurate biogenesis, and CRISPR/Cas induced mutations, such as targeted deletions or base edits, can disrupt these processes to generate mutant lines with varied functions of miRNAs. Such strategies enable the precise functional characterization of stress-responsive miRNAs and their regulatory networks. In millets, where resilience to drought, heat, and poor soils is central to their agronomic value, CRISPR/Cas mediated editing of miRNAs offers a promising strategy to understand and further enhance stress tolerance, yield stability, and nutritional quality. For instance, targeted repression of negative stress regulators or editing of promoter regions to fine tune miRNA expression could accelerate the climate resilience in millet cultivars. While the application of CRISPR/Cas to millet miRNAs is still nascent, integrating this technology with multi omics and breeding approaches will be essential to translate functional insights into practical crop improvement.

CRISPR enables precise manipulation of miRNA biogenesis, promoters, and target sites to decode and deploy stress-response modules in millets. Proof of concept in cereals shows that Cas9 knockouts of miRNA hairpins (single or family-wide) generate null or hypomorphic alleles and reveal roles in development and stress (Chung et al. 2020; Zhou et al. 2017). For translational pipelines in foxtail millet and pearl millet, priorities include (i) deleting drought-susceptibility miRNAs and (ii) engineering promoter cis-elements for high stress inducibility without baseline penalties. Editing miRNA binding sites on targets is especially powerful. In rice, disrupting the miR396 sites in *OsGRF4* and *OsGRF8* derepressed growth, boosting grain size and insect resistance (Lin et al. 2021). In wheat, editing the miR156 MRE in *TaSPL13* improved grain number, size and architecture (Gupta et al. 2023). Together with base editing, such alleles provide tunable miRNA sensitivity responses suited to arid and semi-arid production ecologies.

Emerging evidence implicates specific miRNAs in the control of mineral uptake and transport, including regulatory modules that affect iron and mineral homeostasis in rice (Kehr 2013; Agarwal et al. 2015). Identifying miRNAs that target Fe and Zn transporter genes in millets and experimentally validating those interactions therefore offers a promising route to biofortification. Expression modification strategies such as *miRNA* overexpression, target mimicry, promoter editing, or precise editing of miRNA/target recognition sites (using CRISPR/Cas approaches) can be deployed to upregulate transporter activity or relieve negative regulation, with the ultimate objective of increasing millet grain micronutrient accumulation.

### Artificial miRNAs and target mimics for metabolic reprogramming

Artificial miRNAs (amiRNAs) deliver gene specific knockdown with minimal off targets and can be stacked or driven by tissue-specific promoters(Ossowski et al. 2008; Schwab et al. 2006). Complementarily, miRNA target mimicry, including miRNA mimic (MIM) and short tandem target mimic (STTM) approaches, sequesters endogenous miRNAs, thereby relieving repression of their target genes and elevating the expression of beneficial regulatory factors. Nutrient-responsive axes such as *miR399–PHO2* (Pi efficiency) illustrate how mimics can re-route uptake/remobilization (Hackenberg et al. 2013). In millets, grain specific expression of amiRNAs or mimics can enrich Fe, Zn, and Ca while avoiding vegetative trade-offs, aligning with biofortification targets and yield stability. Strategic combinations, for example, promoter-edited miRNA loci plus endosperm-confined MIMs, offer orthogonal control to decouple stress tolerance from yield and quality penalties. Several studies have demonstrated the utility of amiRNAs in conferring resistance against insect pests. For example, Saini et al. (2018) reported that replacing the Arabidopsis miR164b/miR164b sequence with HaAce1-amiR1, targeting the *acetylcholinesterase1 *(*Ace1*) gene of *Helicoverpa armigera*, in tobacco plants resulted in severe disruption of larval growth and development. Similarly, expression of *amiR-HaEcR* (targeting the ecdysone receptor gene of *H. armigera*) and *amiR-Ace1* (targeting the acetylcholinesterase1 gene of *Myzus persicae*) in tomato led to reduced transcript levels of the respective genes, thereby impairing insect growth and survival (Yogindran and Rajam 2021). Furthermore, Zubair et al. (2020) demonstrated that modification of the miR159 precursor in Arabidopsis to express artificial miRNAs targeting the *sex lethal *(*Sxl*) protein, *acetylcholinesterase *(*AChE*), and *orcokinin *(*Orc*) genes of whitefly effectively reduced infection rates when expressed in tobacco.

### High-throughput screening pipelines for millets

Millets benefit from the *Setaria viridis* (wild foxtail) model, which supports rapid build test learn cycles. The resources are available to cover transformation and phenotyping (Huang et al. 2016), spike dip and shoot apex-based Agrobacterium transformation (Ceasar et al. 2017; Saha and Blumwald 2016) and particle bombardment transients (Mookkan 2018) for efficient mutant line generation for fast CRISPR validation and testing in millets. For accelerated functional screens, foxtail mosaic virus (FoMV) vectors enable virus-induced gene silencing (VIGS) and sgRNA delivery in monocots (Mei et al. 2016). A practical millet pipeline would include (1) designing amiRNA/MIM libraries against drought/heat/nutrient regulators, (2) validating edits and sensitivity tuned alleles in *S. viridis* protoplasts, (3) screening via FoMV-VIGS transient assays, and (4) progressing the best constructs into stable millet genotypes to generate stable mutant lines for multi-environment testing. Linking these edits with phenomics and ionomics will prioritize genotypes that retain farmer-preferred grain quality while delivering climate resilience and micronutrient density. Future miRNA research could focus on combining miRNA gene edits with specially adjusted target genes and testing how stable and reliable these changes are in different environments (G × E) and trials. At the same time, researchers can create tissue-specific artificial miRNA systems, especially in the endosperm, to increase nutrients like iron, zinc, and calcium, without compromising vegetative growth. These gene changes should be combined with advanced breeding methods to speed up improvement. A streamlined testing system, starting with *S. viridis* and moving to other millets (using tools like protoplast assays and quick gene silencing methods), should be set up with clear rules for which changes to keep or drop to make the gene editing process faster. Finally, these genetic changes should be tested in real fields using high-tech plant and nutrient analysis and, where possible, quick miRNA tests. The goal is to create plants that are ready for regulatory approval and fast adoption in countries like India.

## Conclusion

Millets are increasingly recognized as climate-resilient and nutritionally important crops with considerable potential to support sustainable agriculture under changing environmental conditions. Although research on miRNA-mediated regulation in millets remains less developed than in major cereals, accumulating evidence indicates that conserved miRNA families, including *miR156, miR159, miR160, miR167, miR169, miR172, miR393, miR394, miR396, miR39*8, and *miR399*, play important roles in regulating drought adaptation, salinity tolerance, root development, nutrient homeostasis, grain development, and micronutrient accumulation. These findings highlight the central role of miRNA-guided regulatory networks in coordinating stress responses and agronomic traits in millet crops. Despite substantial progress in miRNA identification and expression profiling, experimentally validated regulatory modules remain limited, restricting a comprehensive understanding of their biological functions. Continued advances in functional genomics and integrative systems biology are expected to accelerate the discovery of key miRNA-target networks and their contribution to stress adaptation and nutritional quality. Overall, miRNA research provides a promising foundation for enhancing millet productivity, resilience, and nutritional value, thereby supporting the development of next-generation millet cultivars suited to future agricultural challenges.

## Data Availability

No new datasets were generated or analyzed during the preparation of this manuscript.

## Abbreviations

MiR: MicroRNA
RLM-RACE: RNA Ligase-Mediated Rapid Amplification of cDNA Ends
STTM: Short Tandem Target Mimic
AmiRNA: Artificial miRNAs
CRISPR: Clustered Regularly Interspaced Short Palindromic Repeats
HSP: Heat Shock Protein
SPL: SQUAMOSA promoter binding like
ARF: Auxin response factors
CSD: Copper/zinc superoxide dismutase
CCS: Copper Chaperone for Superoxide Dismutase
HSF: Heat stress transcription factors
CBF: Cold-regulated (CRT/DRE binding factor)
PAMP: Pathogen-associated molecular pattern
NLA: Nitrogen limitation adaptation
GST: Glutathione-S-transferase
ALS: Acetolactate synthase
EPSPS: 5-Enolpyruvylshikimate-3-phosphate synthase
ceRNA: Competing endogenous RNA
AChE: Acetylcholinesterase
Orc: Orcokinin
GRF: Growth regulating factor
TCP: Teosinte branched1 / cycloidea/proliferating cell factor
NRAMP: Natural Resistance-Associated Macrophage Proteins
YSL: Yellow Stripe-Like transporters,
NAS: Nicotianamine Synthase,
FoMV: Foxtail mosaic virus vectors
VIGS: Virus-induced gene silencing

## References

[CR1] Abedin MJ, Abdullah ATM, Satter MA, Farzana T (2022) Physical, functional, nutritional and antioxidant properties of foxtail millet in Bangladesh. Heliyon 8(0):e11186. 10.1016/j.heliyon.2022.e11186 36339997 10.1016/j.heliyon.2022.e11186 PMC9626931

[CR2] Abhigna D, Kalpana R, Radhamani S, Ravichandran V, Janaki P, Geetha P (2024) Performance of Brown top millet (*Brachiaria ramosa* L.) grown under problematic soils. J Appl Nat Sci 16(2):503. 10.31018/jans.v16i2.5471

[CR3] Agarwal S, Mangrauthia SK, Sarla N (2015) Expression profiling of iron deficiency responsive microRNAs and gene targets in rice seedlings of Madhukar x Swarna recombinant inbred lines with contrasting levels of iron in seeds. Plant Soil 396(1):137–150. 10.1007/s11104-015-2561-y

[CR4] Aggarwal PR, Pramitha L, Choudhary P, Singh RK, Shukla P, Prasad M, Muthamilarasan M (2022) Multi-omics intervention in *Setaria* to dissect climate-resilient traits: progress and prospects. Front Plant Sci 13:892736. 10.3389/fpls.2022.892736 36119586 10.3389/fpls.2022.892736 PMC9470963

[CR5] Al-Khayri JM, Jain SM, Johnson DV (eds) (2019) Advances in plant breeding strategies: Cereals. Springer Cham. 10.1007/978-3-030-23108-8

[CR6] Alptekin B, Akpinar BA, Budak H (2017) A comprehensive prescription for plant miRNA identification. Front Plant Sci 7:2058. 10.3389/fpls.2016.02058 28174574 10.3389/fpls.2016.02058 PMC5258749

[CR7] Amtmann A, Blatt MR (2009) Regulation of macronutrient transport. New Phytol 181:5–52. 10.1111/j.1469-8137.2008.02666.x 19076716 10.1111/j.1469-8137.2008.02666.x

[CR8] Anitha S, Kane-Potaka J, Botha R, Givens DI, Sulaiman NLB, Upadhyay S, Vetriventhan M, Tsusaka TW, Parasannanavar DJ, Longvah T (2021a) Millets can have a major impact on improving iron status, hemoglobin level, and in reducing iron deficiency anemia–a systematic review and meta-analysis. Front Nutr 8:725529. 10.3389/fnut.2021.725529 34722606 10.3389/fnut.2021.725529 PMC8551390

[CR9] Anitha S, Kane-Potaka J, Tsusaka TW, Botha R, Rajendran A, Givens DI, Parasannanavar DJ, Subramaniam K, Prasad KDV, Vetriventhan M (2021b) A systematic review and meta-analysis of the potential of millets for managing and reducing the risk of developing diabetes mellitus. Front Nutr 8:687428. 10.3389/fnut.2021.687428 34395493 10.3389/fnut.2021.687428 PMC8355360

[CR10] Anitha S, Tsusaka TW, Givens DI, Kane-Potaka J, Botha R, Sulaiman NLB, Upadhyay S, Vetriventhan M, Rajendran A, Parasannanavar DJ (2024) Does millet consumption contribute to raising blood hemoglobin levels compared to regular refined staples?: a systematic review and meta-analysis. Front Nutr 11:1305394. 10.3389/fnut.2024.1305394 38419846 10.3389/fnut.2024.1305394 PMC10900984

[CR11] Aslam M, Sugita K, Qin Y, Rahman A (2020) Aux/IAA14 regulates microRNA-mediated cold stress response in *Arabidopsis* roots. Int J Mol Sci 21(22):8441. 10.3390/ijms21228441 33182739 10.3390/ijms21228441 PMC7697755

[CR12] Assefa K, Chanyalew S, Tadele Z (2017) Tef, Eragrostis tef (Zucc.) Trotter. In Millets and Sorghum, J.V. Patil (Ed.). 10.1002/9781119130765.ch9

[CR13] Baek D, Chun HJ, Kang S, Shin G, Park SJ, Hong H, Kim C, Kim DH, Lee SY, Kim MC, Yun D-J (2016) A role for *Arabidopsis* miR399f in salt, drought, and ABA signaling. Mol Cells 39(2):111–118. 10.14348/molcells.2016.2188 26674968 10.14348/molcells.2016.2188 PMC4757798

[CR14] Bandyopadhyay T, Muthamilarasan M, Prasad M (2017) Millets for next generation climate-smart agriculture. Front Plant Sci 8:1266. 10.3389/fpls.2017.01266 28769966 10.3389/fpls.2017.01266 PMC5513978

[CR15] Barciszewska-Pacak M, Milanowska K, Knop K, Bielewicz D, Nuc P, Plewka P, Pacak AM, Vazquez F, Karlowski W, Jarmolowski A, Szweykowska-Kulinska Z (2015) Arabidopsis microRNA expression regulation in a wide range of abiotic stress responses. Front Plant Sci 6:410. 10.3389/fpls.2015.00410 10.3389/fpls.2015.00410 PMC4454879 26089831

[CR16] Bari R, Datt Pant B, Stitt M, Scheible WR (2006) PHO2, microRNA399, and PHR1 define a phosphate-signaling pathway in plants. Plant Physiol 141(3):988–999. 10.1104/pp.106.079707 16679424 10.1104/pp.106.079707 PMC1489890

[CR17] Barretto R, Buenavista RM, Rivera JL, Wang S, Prasad PVV, Siliveru K (2021) Teff (*Eragrostis tef*) processing, utilization and future opportunities: a review. Int J Food Sci Technol 56(7):3125–3137. 10.1111/ijfs.14872

[CR18] Bej S, Basak J (2014) MicroRNAs: the potential biomarkers in plant stress response. Am J Plant Sci 5:748–759. 10.4236/ajps.2014.55089

[CR19] Bhavani P, Nandini C, Maharajan T, Ningaraju TM, Nandini B, Parveen SG, Pushpa K, Ravikumar RL, Nagaraja TE, Ceasar SA (2024) Brown-top millet: an overview of breeding, genetic, and genomic resources development for crop improvement. Planta 260(1):10. 10.1007/s00425-024-04446-7 38796805 10.1007/s00425-024-04446-7

[CR20] Boccara M, Sarazin A, Thiébeauld O, Jay F, Voinnet O, Navarro L, Colot V (2015) Correction: The *Arabidopsis* miR472-RDR6 silencing pathway modulates PAMP- and effector-triggered immunity through the post-transcriptional control of disease resistance genes. PLoS Pathog 11(4):e1004814. 10.1371/journal.ppat.1004814 25859662 10.1371/journal.ppat.1004814 PMC4393137

[CR21] Bonnet E, Wuyts J, Rouzé P, Van de Peer Y (2004) Detection of 91 potential conserved plant microRNAs in *Arabidopsis thaliana* and *Oryza sativa* identifies important target genes. Proc Natl Acad Sci U S A 101(31):11511–11516. 10.1073/pnas.0404025101 15272084 10.1073/pnas.0404025101 PMC509231

[CR22] Borrell A, Jordan D, Mullet J, Henzell B, Hammer G (2024) Drought adaptation in sorghum. In: Drought adaptation in cereals. CRC Press, pp 335–399. 10.1201/9781003578338-13

[CR23] Cai H, Lu Y, Xie W, Zhu T, Lian X (2012) Transcriptome response to nitrogen starvation in rice. J Biosci 37(4):731–747. 10.1007/s12038-012-9242-2 22922198 10.1007/s12038-012-9242-2

[CR24] Cannarozzi G, Tadele Z (2022) Tef [*Eragrostis tef* (Zucc.) Trotter]. In: Chapman MA (eds) Underutilised Crop Genomes. Compendium of Plant Genomes. Springer, Cham. 10.1007/978-3-031-00848-1_3

[CR25] Ceasar SA, Baker A (2025) Millets for food security and agricultural sustainability. Planta 261(2):39. 10.1007/s00425-025-04613-4 39812887 10.1007/s00425-025-04613-4

[CR26] Ceasar SA, Baker A, Ignacimuthu S (2017) Functional characterization of the PHT1 family transporters of foxtail millet with development of a novel *Agrobacterium*-mediated transformation procedure. Sci Rep 7(1):14064. 10.1038/s41598-017-14447-0 29070807 10.1038/s41598-017-14447-0 PMC5656669

[CR27] Chakraborty A, Viswanath A, Malipatil R, Rathore A, Thirunavukkarasu N (2020) Structural and Functional Characteristics of miRNAs in Five Strategic Millet Species and Their Utility in Drought Tolerance. Front Genet 11:608421. 10.3389/fgene.2020.608421 10.3389/fgene.2020.608421 PMC7753210 33363575

[CR28] Chandra AK, Pandey D, Sood S, Joshi DC, Tiwari A, Sharma D, Gururani K, Kumar A (2024) Uncovering the genomic regions underlying grain iron and zinc content using genome-wide association mapping in finger millet. 3 Biotech 14(2):47. 10.1007/s13205-023-03889-1 38268987 10.1007/s13205-023-03889-1 PMC10803704

[CR29] Chanyalew S, Ferede S, Damte T, Fikre T, Genet Y, Kebede W, Tolossa K, Tadele Z, Assefa K (2019) Significance and prospects of an orphan crop tef. Planta 250(3):753–767. 10.1007/s00425-019-03209-z 31222492 10.1007/s00425-019-03209-z

[CR30] Chen X (2009) Small RNAs and their roles in plant development. Annu Rev Cell Dev Biol 25(1):21–44. 10.1146/annurev.cellbio.042308.113417 19575669 10.1146/annurev.cellbio.042308.113417 PMC5135726

[CR31] Chung PJ, Chung H, Oh N, Choi J, Bang SW, Jung SE, Jung H, Shim JS, Kim JK (2020) Efficiency of recombinant CRISPR/rCas9-mediated miRNA gene editing in rice. Int J Mol Sci 21(24):9606. 10.3390/ijms21249606 33339449 10.3390/ijms21249606 PMC7766165

[CR32] Cui LG, Shan JX, Shi M, Gao JP, Lin HX (2014) The miR156-SPL9-DFR pathway coordinates the relationship between development and abiotic stress tolerance in plants. Plant J 80(6):1108–1117. 10.1111/tpj.12712 25345491 10.1111/tpj.12712

[CR33] Curaba J, Singh MB, Bhalla PL (2014) MiRNAs in the crosstalk between phytohormone signaling pathways. J Exp Bot 65(6):1425–1438. 10.1093/jxb/eru002 24523503 10.1093/jxb/eru002

[CR34] Das S, Khound R, Santra M, Santra DK (2019) Beyond bird feed: proso millet for human health and environment. Agriculture 9(3):64. 10.3390/agriculture9030064

[CR35] Deepak Taggelli RG, Thakur V (2018) Minor millets-their potential health benefits and medicinal properties: A review. Int J Pure Appl Sci 6(1):1677. 10.18782/2320-7051.6466

[CR36] Deng Y, Zhang H, Wang H, Xing G, Lei B, Kuang Z, Zhao Y, Li C, Dai S, Yang X, Wei J, Zhang J (2022). The Construction and Exploration of a Comprehensive MicroRNA Centered Regulatory Network in Foxtail Millet (*Setaria italica* L.). Front Plant Sci *13:*848474. 10.3389/FPLS.2022.848474/BIBTEX 10.3389/fpls.2022.848474 PMC9121102 35599893

[CR37] Denver JB, Ullah H (2019) MiR393s regulate salt stress response pathway in *Arabidopsis thaliana* through scaffold protein RACK1A mediated ABA signaling pathways. Plant Signal Behav 14(6):1600394. 10.1080/15592324.2019.1600394 31021701 10.1080/15592324.2019.1600394 PMC6546147

[CR38] Department of Biotechnology India (2022) Guidelines for the Safety Assessment of Genome Edited Plants 2022. Ministry of Science and Technology, Government of India

[CR39] Dey S, Saxena A, Kumar Y, Maity T, Tarafdar A (2022) Understanding the Antinutritional Factors and Bioactive Compounds of Kodo Millet (*Paspalum scrobiculatum*) and Little Millet (*Panicum sumatrense*). J Food Qual 2022:1578448. 10.1155/2022/1578448

[CR40] Ding X, Guo J, Zhang Q, Yu L, Zhao T, Yang S (2021) Heat-responsive miRNAs participate in the regulation of male fertility stability in soybean CMS-based F1 under high temperature stress. Int J Mol Sci 22(5):2446. 10.3390/ijms22052446 33671046 10.3390/ijms22052446 PMC7957588

[CR41] Djami-Tchatchou AT, Sanan-Mishra N, Ntushelo K, Dubery IA (2017) Functional roles of microRNAs in agronomically important plants-potential as targets for crop improvement and protection. Front Plant Sci 8:378. 10.3389/FPLS.2017.00378, 10.3389/fpls.2017.00378 PMC5360763 28382044

[CR42] Dong CH, Pei H (2014) Over-expression of miR397 improves plant tolerance to cold stress in *Arabidopsis thaliana*. J Plant Biol 57(4):209–217. 10.1007/s12374-013-0490-y

[CR43] Dong Q, Hu B, Zhang C (2022) Micrornas and their roles in plant development. Front Plant Sci 13:824240. 10.3389/fpls.2022.824240 35251094 10.3389/fpls.2022.824240 PMC8895298

[CR44] Fasani E, DalCorso G, Zorzi G, Vitulo N, Furini A (2021) Comparative analysis identifies micro-RNA associated with nutrient homeostasis, development and stress response in *Arabidopsis thaliana* upon high Zn and metal hyperaccumulator *Arabidopsis halleri*. Physiol Plant 173(3):920–934. 10.1111/ppl.13488 34171137 10.1111/ppl.13488 PMC8597110

[CR45] Ferdous J, Hussain SS, Shi B (2015) Role of micro RNAs in plant drought tolerance. Plant Biotechnol J 13(3):293–305. 10.1111/pbi.12318 25583362 10.1111/pbi.12318 PMC6680329

[CR46] Flores CI, Clark RB, Gourley LM (1991) Genotypic variation of pearl millet for growth and yield on acid soil. Field Crops Res 26(3):347–354. 10.1016/0378-4290(91)90010-S

[CR47] Fu D, Ma BI, Mason AS, Xiao M, Wei L, An Z (2013) Micro RNA‐based molecular markers: a novel PCR‐based genotyping technique in *Brassica* species. Plant Breed 132(4):375–381. 10.1111/pbr.12069

[CR48] Fu F, Girma G, Mengiste T (2020) Global mRNA and microRNA expression dynamics in response to anthracnose infection in sorghum. BMC Genomics 21(1):760. 10.1186/s12864-020-07138-0 33143636 10.1186/s12864-020-07138-0 PMC7641857

[CR49] Fukuda M, Fujiwara T, Nishida S (2020) Roles of non-coding RNAs in response to nitrogen availability in plants. Int J Mol Sci 21(22):8508. 10.3390/ijms21228508 33198163 10.3390/ijms21228508 PMC7696010

[CR50] Fuyou, Fu Gezahegn, Girma Tesfaye, Mengiste (2020) Global mRNA and microRNA expression dynamics in response to anthracnose infection in sorghum. BMC Genomics 21(1):760. 10.1186/s12864-020-07138-0 10.1186/s12864-020-07138-0 PMC7641857 33143636

[CR51] Gangadhar BH, Venkidasamy B, Samynathan R, Saranya B, Chung IM, Thiruvengadam M (2021) Overview of miRNA biogenesis and applications in plants. Biologia (Bratisl) 76(8):2309–2327. 10.1007/s11756-021-00763-4

[CR52] Gangashetty PI, Riyazaddin M, Sanogo MD, Inousa D, Issoufou KA, Asungre PA, Sy O, Govindaraj M, Ignatius AI (2021) Identification of high-yielding iron-biofortified open-pollinated varieties of pearl millet in West Africa. Front Plant Sci 12:688937. 10.3389/fpls.2021.688937 34630450 10.3389/fpls.2021.688937 PMC8492886

[CR53] Gao P, Bai X, Yang L, Lv D, Pan X, Li Y, Cai H, Ji W, Chen Q, Zhu Y (2011) *osa*-MIR393: a salinity- and alkaline stress-related microRNA gene. Mol Biol Rep 38(1):237–242. 10.1007/s11033-010-0100-8 20336383 10.1007/s11033-010-0100-8

[CR54] Gao S, Guo C, Zhang Y, Zhang F, Du X, Gu J, Xiao K (2016) Wheat microRNA member TaMIR444a is nitrogen deprivation-responsive and involves plant adaptation to the nitrogen-starvation stress. Plant Mol Biol Rep 34(5):931–946. 10.1007/s11105-016-0973-3

[CR55] Geng Z, Liu J, Li D, Zhao G, Liu X, Dou H, Lv L, Zhang H, Wang Y (2021) A conserved miR394-targeted F-box gene positively regulates drought resistance in foxtail millet. J Plant Biol 64(3):243–252. 10.1007/s12374-021-09303-8

[CR56] Ghodoum Parizipour M, Tahmasebi A, Shahriari AG (2025) Computational analysis of microRNAs in sweet sorghum (*Sorghum bicolor*) infected by Sorghum Mosaic Virus. Sugar Tech 27(6):1796–1805. 10.1007/s12355-025-01622-1

[CR57] Govindaraj M, Rai KN, Cherian B, Pfeiffer WH, Kanatti A, Shivade H (2019) Breeding biofortified pearl millet varieties and hybrids to enhance millet markets for human nutrition. Agriculture 9(5):106. 10.3390/agriculture9050106

[CR58] Gowda NAN, Siliveru K, Prasad PVV, Bhatt Y, Netravati BP, Gurikar C (2022) Modern processing of Indian millets: a perspective on changes in nutritional properties. Foods 11(4):499. 10.3390/foods11040499 35205975 10.3390/foods11040499 PMC8871339

[CR59] Grennan AK (2008) Phosphate accumulation in plants: signaling. Plant Physiol 148(1):3–5. 10.1104/pp.104.900269 18772350 10.1104/pp.104.900269 PMC2528084

[CR60] Guan Q, Lu X, Zeng H, Zhang Y, Zhu J (2013) Heat stress induction of *miR398* triggers a regulatory loop that is critical for thermotolerance in Arabidopsis. Plant J 74(5):840–851. 10.1111/tpj.12169 23480361 10.1111/tpj.12169

[CR61] Guo S, Xu Y, Liu H, Mao Z, Zhang C, Ma Y, Zhang Q, Meng Z, Chong K (2013) The interaction between OsMADS57 and OsTB1 modulates rice tillering via DWARF14. Nat Commun 4(1):1566. 10.1038/ncomms2542 23463009 10.1038/ncomms2542 PMC3615354

[CR62] Gupta A, Patil M, Qamar A, Senthil-Kumar M (2020) Ath-miR164c influences plant responses to the combined stress of drought and bacterial infection by regulating proline metabolism. Environ Exp Bot 172:103998. 10.1016/j.envexpbot.2020.103998

[CR63] Gupta A, Hua L, Zhang Z, Yang B, Li W (2023) CRISPR-induced miRNA156-recognition element mutations in TaSPL13 improve multiple agronomic traits in wheat. Plant Biotechnol J 21(3):536–548. 10.1111/pbi.13969 36403232 10.1111/pbi.13969 PMC9946137

[CR64] Habte ML, Beyene EA, Feyisa TO, Admasu FT, Tilahun A, Diribsa GC (2022) Nutritional values of teff (*Eragrostis tef*) in diabetic patients: narrative review. Diabetes Metab Syndr Obes 15:2599–2606. 10.2147/DMSO.S366958 36035517 10.2147/DMSO.S366958 PMC9416382

[CR65] Hackenberg M, Shi B-J, Gustafson P, Langridge P (2013) Characterization of phosphorus-regulated miR399 and miR827 and their isomirs in barley under phosphorus-sufficient and phosphorus-deficient conditions. BMC Plant Biol 13(1):214. 10.1186/1471-2229-13-214 24330740 10.1186/1471-2229-13-214 PMC3878733

[CR66] Hamza NB, Sharma N, Tripathi A, Sanan-Mishra N (2016) MicroRNA expression profiles in response to drought stress in *Sorghum bicolor*. Gene Expr Patterns 20(2):88–98. 10.1016/j.gep.2016.01.001 26772909 10.1016/j.gep.2016.01.001

[CR67] Han J, Xie H, Sun Q, Wang J, Lu M, Wang W, Guo E, Pan J (2014) Bioinformatic identification and experimental validation of miRNAs from foxtail millet (*Setaria italica*). Gene 546(2):367–377. 10.1016/j.gene.2014.05.050 24862217 10.1016/j.gene.2014.05.050

[CR68] Han Y, Zhang X, Wang W, Wang Y, Ming F (2015) Correction: the suppression of WRKY44 by GIGANTEA-miR172 pathway is involved in drought response of *Arabidopsis thaliana*. PLoS ONE 10(4):e0124854. 10.1371/journal.pone.0124854 25844872 10.1371/journal.pone.0124854 PMC4386900

[CR69] Hassan ZM, Sebola NA, Mabelebele M (2021) The nutritional use of millet grain for food and feed: a review. Agric Food Secur 10(1):16. 10.1186/s40066-020-00282-6 33815778 10.1186/s40066-020-00282-6 PMC8005370

[CR70] Hemasai B, Kumbha DK, Modem VN, Gannavarapu SK, Bommaka RR, Mallapuram S, Chintala S, Sreevalli MD, Ramireddy E, Vemireddy LR (2024) Development of miRNA-SSR and target-SSR markers from yield-associate genes and their applicability in the assessment of genetic diversity and association mapping in rice (*Oryza sativa* L.). Mol Breed 44(4):30. 10.1007/S11032-024-01462-Z 38634111 10.1007/s11032-024-01462-z PMC11018576

[CR71] Hou S, Men Y, Zhang Y, Zhao K, Ma G, Li H, Han Y, Sun Z (2022) Role of miRNAs in regulation of SA-mediated upregulation of genes involved in folate and methionine metabolism in foxtail millet. Front Plant Sci 13:1023764. 10.3389/fpls.2022.1023764 36561440 10.3389/fpls.2022.1023764 PMC9763449

[CR72] Hu H, Mauro-Herrera M, Doust AN (2018) Domestication and improvement in the model C4 grass, Setaria. Front Plant Sci 9:719. 10.3389/fpls.2018.00719 29896214 10.3389/fpls.2018.00719 PMC5986938

[CR73] Huang P, Shyu C, Coelho CP, Cao Y, Brutnell TP (2016) *Setaria viridis* as a model system to advance millet genetics and genomics. Front Plant Sci 7:1781. 10.3389/fpls.2016.01781 10.3389/fpls.2016.01781 PMC5124564 27965689

[CR74] Huen AK, Rodriguez‐Medina C, Ho AYY, Atkins CA, Smith PMC (2017) Long‐distance movement of phosphate starvation‐responsive microRNAs in *Arabidopsis*. Plant Biol (Stuttg) 19(4):643–649. 10.1111/plb.12568 10.1111/plb.12568 28322489

[CR75] Ibrahim Bio Yerima AR, Achigan‐Dako EG (2021) A review of the orphan small grain cereals improvement with a comprehensive plan for genomics‐assisted breeding of fonio millet in West Africa. Plant Breed 140(4):561–574. 10.1111/pbr.12930

[CR76] Iglesias MJ, Terrile MC, Windels D, Lombardo MC, Bartoli CG, Vazquez F, Estelle M, Casalongué CA (2014) MiR393 regulation of auxin signaling and redox-related components during acclimation to salinity in *Arabidopsis*. PLoS ONE 9(9):e107678. 10.1371/journal.pone.0107678 25222737 10.1371/journal.pone.0107678 PMC4164656

[CR77] Indirani K (2021) Review on nutritional profiles and health benefits of little millets–India. Int J Res Eng Sci 9(11):7–11

[CR78] Islam W, Waheed A, Naveed H, Zeng F (2022) MicroRNAs mediated plant responses to salt stress. Cells 11(18):1–22. 10.3390/cells11182806 10.3390/cells11182806 PMC9496875 36139379

[CR79] Jagadeesh Selvam HRN, Senthil N, Manikanda Boopathi N, Raveendran M (2015) Computational identification of salinity responsive microRNAs in contrasting genotypes of Finger millet (*Eleusine coracana* L.). Res J Biotechnol 10(9):52–64.

[CR80] Jiao Y, Wang Y, Xue D, Wang J, Yan M, Liu G, Dong G, Zeng D, Lu Z, Zhu X (2010) Regulation of OsSPL14 by OsmiR156 defines ideal plant architecture in rice. Nat Genet 42(6):541–544. 10.1038/ng.591 20495565 10.1038/ng.591

[CR81] James JW, Antle JM, Basso B, Boote KJ, Conant RT, Foster I, Godfray HCJ, Herrero M, Howitt RE, Janssen S, Keating BA, Munoz-Carpena R, Porter CH, Rosenzweig C, Wheeler TR (2017) Toward a new generation of agricultural system data, models, and knowledge products: State of agricultural systems science. Agric Syst 155:269–288. 10.1016/j.agsy.2016.09.021 10.1016/j.agsy.2016.09.021 PMC5485672 28701818

[CR82] Jung HJ, Kang H (2007) Expression and functional analyses of microRNA417 in *Arabidopsis thaliana* under stress conditions. Plant Physiol Biochem 45(10–11):805–811. 10.1016/j.plaphy.2007.07.015 17845858 10.1016/j.plaphy.2007.07.015

[CR83] Karkada S, Upadhya S, Upadhya S, Bhat G (2019) Beneficial effects of ragi (finger millet) on hematological parameters, body mass index, and scholastic performance among anemic adolescent high-school girls (AHSG). Compr Child Adolesc Nurs 42(2):141–150. 10.1080/24694193.2018.1440031 29595341 10.1080/24694193.2018.1440031

[CR84] Kasanaboina K, Ishwarya Lakshmi VG, Nandigam SR, Jagadeesh K, Ceasar SA (2025) The role of millets in climate-resilient agriculture for conserving global food security amidst climate change. Nucleus. 10.1007/s13237-025-00599-2

[CR85] Katiyar A, Smita S, Muthusamy SK, Chinnusamy V, Pandey DM, Bansal KC (2015) Identification of novel drought-responsive microRNAs and trans-acting siRNAs from *Sorghum bicolor* (L.) Moench by high-throughput sequencing analysis. Front Plant Sci 6:506. 10.3389/fpls.2015.00506 10.3389/fpls.2015.00506 PMC4504434 26236318

[CR86] Kehr J (2013) Systemic regulation of mineral homeostasis by micro RNAs. Front Plant Sci 4:145. 10.3389/fpls.2013.00145 23720667 10.3389/fpls.2013.00145 PMC3655319

[CR87] Khraiwesh B, Zhu J-K, Zhu J (2012) Role of miRNAs and siRNAs in biotic and abiotic stress responses of plants. Biochim Biophys Acta 1819(2):137–148. 10.1016/j.bbagrm.2011.05.001 21605713 10.1016/j.bbagrm.2011.05.001 PMC3175014

[CR88] Kumar V, Yadav M, Awala SK, Valombola JS, Saxena MS, Ahmad F, Saxena SC (2024) Millets: a nutritional powerhouse for ensuring food security. Planta 260(4):101. 10.1007/s00425-024-04533-9 39302511 10.1007/s00425-024-04533-9

[CR89] Kumar A, Tomer V, Kaur A, Kumar V, Gupta K (2018) Millets: A solution to agrarian and nutritional challenges. Agric Food Secur 7:31. 10.1186/s40066-018-0183-3

[CR90] Kumari A, Sadh PK, Kamboj A, Yadav B, Kumar A, Sivakumar S, Saharan BS, Brar B, Goyal C (2025) Exploring the Benefits of Nutrition of Little Millet: Unveiling the Effect of Processing Methods on Bioactive Properties. J Food Biochem 2025(1):2488816. 10.1155/jfbc/2488816

[CR91] Lee B, Henderson DA, Zhu JK (2005) The *Arabidopsis* cold-responsive transcriptome and its regulation by ICE1. Plant Cell 17(11):3155–3175. 10.1105/tpc.105.035568 16214899 10.1105/tpc.105.035568 PMC1276035

[CR92] Li C, Zhang B (2016) MicroRNAs in control of plant development. J Cell Physiol 231(2):303–313. 10.1002/jcp.25125 26248304 10.1002/jcp.25125

[CR93] Li WX, Oono Y, Zhu J, He XJ, Wu JM, Iida K, Lu XY, Cui X, Jin H, Zhu JK (2008) The *Arabidopsis* NFYA5 transcription factor is regulated transcriptionally and posttranscriptionally to promote drought resistance. Plant Cell 20(8):2238–2251. 10.1105/tpc.108.059444 18682547 10.1105/tpc.108.059444 PMC2553615

[CR94] Li W, Wang T, Zhang Y, Li Y (2016) Overexpression of soybean miR172c confers tolerance to water deficit and salt stress, but increases ABA sensitivity in transgenic *Arabidopsis thaliana*. J Exp Bot 67(1):175–194. 10.1093/jxb/erv450 26466661 10.1093/jxb/erv450

[CR95] Li Q, Li D, Guo S, Yu X (2024) Genome-Wide Identification of microRNAs Associated with Starch Biosynthesis and Endosperm Development in Foxtail Millet. Int J Mol Sci 25(17):9282. 10.3390/IJMS25179282/S1 39273232 10.3390/ijms25179282 PMC11395324

[CR96] Lian JP, Yuan C, Feng YZ, Liu Q, Wang CY, Zhou YF, Huang QJ, Zhu QF, Zhang YC, Chen YQ, Yu Y (2024) MicroRNA397 promotes rice flowering by regulating the photorespiration pathway. Plant Physiol 194(4):2101–2116. 10.1093/PLPHYS/KIAD626 37995372 10.1093/plphys/kiad626

[CR97] Liang G, He H, Yu D (2012) Identification of nitrogen starvation-responsive microRNAs in *Arabidopsis thaliana*. PLoS ONE 7(11):e48951. 10.1371/journal.pone.0048951 10.1371/journal.pone.0048951 PMC3498362 23155433

[CR98] Lin WY, Huang TK, Chiou TJ (2013) Nitrogen limitation adaptation, a target of microRNA827, mediates degradation of plasma membrane–localized phosphate transporters to maintain phosphate homeostasis in *Arabidopsis*. Plant Cell 25(10):4061–4074. 10.1105/tpc.113.116012 10.1105/tpc.113.116012 PMC3877804 24122828

[CR99] Lin Y, Zhu Y, Cui Y, Chen R, Chen Z, Li G, Fan M, Chen J, Li Y, Guo X, Zheng X, Chen L, Wang F (2021) Derepression of specific miRNA-target genes in rice using CRISPR/Cas9. J Exp Bot 72(20):7067. 10.1093/JXB/ERAB336 10.1093/jxb/erab336 PMC8547147 34283216

[CR100] Lin JS, Kuo CC, Yang IC, Tsai WA, Shen YH, Lin CC, Liang YC, Li YC, Kuo YW, King YC, Lai HM, Jeng ST (2018) MicroRNA160 Modulates Plant Development and Heat Shock Protein Gene Expression to Mediate Heat Tolerance in Arabidopsis. Front Plant Sci 9:68. 10.3389/fpls.2018.00068 10.3389/fpls.2018.00068 PMC5799662 29449855

[CR101] Liu P, Montgomery TA, Fahlgren N, Kasschau KD, Nonogaki H, Carrington JC (2007) Repression of AUXIN RESPONSE FACTOR10 by microRNA160 is critical for seed germination and post-germination stages. Plant J 52(1):133–146. 10.1111/j.1365-313X.2007.03218.x 10.1111/j.1365-313X.2007.03218.x 17672844

[CR102] Longvah T, Ananthan R, Bhaskarachary K, Venkaiah K (2017) Indian Food Composition Tables

[CR103] Loreti E, Perata P (2022) Mobile plant microRNAs allow communication within and between organisms. New Phytol 235(6):2176–2182. 10.1111/nph.18360 35794849 10.1111/nph.18360 PMC10114960

[CR104] Lu G, Gao C, Zheng X, Han B (2009) Identification of OsbZIP72 as a positive regulator of ABA response and drought tolerance in rice. Planta 229(3):605–615. 10.1007/s00425-008-0857-3 10.1007/s00425-008-0857-3 19048288

[CR105] Lundgren MR, Dunning LT, Olofsson JK, Moreno‐Villena JJ, Bouvier JW, Sage TL, Khoshravesh R, Sultmanis S, Stata M, Ripley BS (2019) C4 anatomy can evolve via a single developmental change. Ecol Lett 22(2):302–312. 10.1111/ele.13191 30557904 10.1111/ele.13191 PMC6849723

[CR106] Lydia Pramitha J, Ganesan J, Francis N, Rajasekharan R, Thinakaran J (2023) Revitalization of small millets for nutritional and food security by advanced genetics and genomics approaches. Front Genet 13:1007552. 10.3389/fgene.2022.1007552 36699471 10.3389/fgene.2022.1007552 PMC9870178

[CR107] Lydia Pramitha J, Subalakshmi S, Jeeva G, Surendhar A (2021) Small millets exploring the hidden potential. Scripown Publications, New Delhi

[CR108] Ma C, Burd S, Lers A (2015) *miR 408* is involved in abiotic stress responses in Arabidopsis. Plant J 84(1):169–187. 10.1111/tpj.12999 10.1111/tpj.12999 26312768

[CR109] Maharajan T, Antony Ceasar S, Ajeesh Krishna TP, Ignacimuthu S (2021) Finger millet [*Eleusine coracana* (L.) Gaertn]: an orphan crop with a potential to alleviate the calcium deficiency in the semi-arid tropics of Asia and Africa. Front Sustain Food Syst 5:684447. 10.3389/fsufs.2021.684447

[CR110] Maharajan T, Krishna TPA, Krishnakumar NM, Vetriventhan M, Kudapa H, Ceasar SA (2024) Role of genome sequences of major and minor millets in strengthening food and nutritional security for future generations. Agriculture 14(5):670. 10.3390/agriculture14050670

[CR111] Mahendrakar MD, Parveda M, Kishor PBK, Srivastava RK (2020) Discovery and validation of candidate genes for grain iron and zinc metabolism in pearl millet [*Pennisetum glaucum* (L.) R. Br.]. Sci Rep 10(1):16562. 10.1038/s41598-020-73241-7 33024155 10.1038/s41598-020-73241-7 PMC7538586

[CR112] Mamidi S, Healey A, Huang P, Grimwood J, Jenkins J, Barry K, Sreedasyam A, Shu S, Lovell JT, Feldman M, Wu J, Yu Y, Chen C, Johnson J, Sakakibara H, Kiba T, Sakurai T, Tavares R, Nusinow DA, Baxter I, Schmutz J, Brutnell TP, Kellogg EA (2020) A genome resource for green millet Setaria viridis enables discovery of agronomically valuable loci. Nat Biotechnol 38(10):1203–1210. 10.1038/s41587-020-0681-2 10.1038/s41587-020-0681-2 PMC7536120 33020633

[CR113] Mandal K, Boro P, Chattopadhyay S (2021) Micro-RNA based gene regulation: a potential way for crop improvements. Plant Gene 27:100312. 10.1016/j.plgene.2021.100312

[CR114] Mao YB, Liu YQ, Chen DY, Chen FY, Fang X, Hong GJ, Wang LJ, Wang JW, Chen XY (2017) Jasmonate response decay and defense metabolite accumulation contributes to age-regulated dynamics of plant insect resistance. Nat Commun 8(1):13925. 10.1038/ncomms13925 28067238 10.1038/ncomms13925 PMC5233801

[CR115] Medina J, Catalá R, Salinas J (2011) The CBFs: three *Arabidopsis* transcription factors to cold acclimate. Plant Sci 180(1):3–11. 10.1016/j.plantsci.2010.06.019 21421341 10.1016/j.plantsci.2010.06.019

[CR116] Mei Y, Zhang C, Kernodle BM, Hill JH, Whitham SA (2016) A Foxtail mosaic virus vector for virus-induced gene silencing in maize. Plant Physiol 171(2):760–772. 10.1104/pp.16.00172 27208311 10.1104/pp.16.00172 PMC4902600

[CR117] Mihrete TB, Mihretu FB (2025) Crop diversification for ensuring sustainable agriculture, risk management and food security. Glob Chall 9(2):2400267. 10.1002/gch2.202400267 39925666 10.1002/gch2.202400267 PMC11802337

[CR118] Mirza N, Marla SS (2019) Finger millet (*Eleusine coracana* L. Gartn.) breeding. In: Al-Khayri J et al (eds) Advances in plant breeding strategies: Cereals. Springer Cham, pp 83–132. 10.1007/978-3-030-23108-8_3

[CR119] Miura K, Ikeda M, Matsubara A, Song XJ, Ito M, Asano K, Matsuoka M, Kitano H, Ashikari M (2010) *OsSPL14* promotes panicle branching and higher grain productivity in rice. Nat Genet 42(6):545–549. 10.1038/ng.592 10.1038/ng.592 20495564

[CR120] Mohanapriya B, Shanmugam A, Francis N, Indhu SM, Ravikesavan R (2024) Breeding barnyard millet for abiotic stress tolerance. In: Mishra S (eds) Genetic improvement of Small Millets. Springer, Singapore. 10.1007/978-981-99-7232-6_24

[CR121] Mondal TK, Ganie SA (2014) Identification and characterization of salt responsive miRNA-SSR markers in rice (*Oryza sativa*). Gene 535(2):204–209. 10.1016/J.GENE.2013.11.033 24315823 10.1016/j.gene.2013.11.033

[CR122] Mookkan M (2018) Particle bombardment–mediated gene transfer and GFP transient expression in *Setaria viridis*. Plant Signal Behav 13(4):e1441657. 10.1080/15592324.2018.1441657 10.1080/15592324.2018.1441657 PMC5933905 29621423

[CR123] Muhammad Aslam M, Waseem M, Jakada BH, Okal EJ, Lei Z, Saqib HSA, Yuan W, Xu W, Zhang Q (2022) Mechanisms of abscisic acid-mediated drought stress responses in plants. Int J Mol Sci 23(3):1084. 10.3390/ijms23031084 35163008 10.3390/ijms23031084 PMC8835272

[CR124] Mukesh Sankar S, Tara Satyavathi C, Barthakur S, Singh SP, Bharadwaj C, Soumya SL (2021) Differential modulation of heat-inducible genes across diverse genotypes and molecular cloning of a sHSP from pearl millet [*Pennisetum glaucum* (L.) R. Br.]. Front Plant Sci 12:659893. 10.3389/fpls.2021.659893 10.3389/fpls.2021.659893 PMC8324246 34335644

[CR125] Mukherjee B, Jha RK, Sattar A, Dutta S, Bhattacharya U, Samanta S, Huirem B, Singh SK, Das S, Bal SK (2025) Harnessing the potential of millets for climate-resilient and sustainable agriculture. Front Plant Sci 16:1574699. 10.3389/fpls.2025.1574699 10.3389/fpls.2025.1574699 PMC12188307 40567416

[CR126] Mundia CW, Secchi S, Akamani K, Wang G (2019) A regional comparison of factors affecting global sorghum production: the case of North America, Asia and Africa’s Sahel. Sustainability 11(7):2135. 10.3390/su11072135

[CR127] Mushtaq NU, Saleem S, Rasool A, Shah WH, Hakeem KR, Rehman RU (2021) Salt stress threshold in millets: perspective on cultivation on marginal lands for biomass. Phyton 90(1):51. 10.32604/phyton.2020.012163

[CR128] Nguyen GN, Rothstein SJ, Spangenberg G, Kant S (2015) Role of microRNAs involved in plant response to nitrogen and phosphorous limiting conditions. Front Plant Sci 6:629. 10.3389/fpls.2015.00629 26322069 10.3389/fpls.2015.00629 PMC4534779

[CR129] Ni Z, Hu Z, Jiang Q, Zhang H (2012) Overexpression of gma-MIR394a confers tolerance to drought in transgenic *Arabidopsis thaliana*. Biochem Biophys Res Commun 427(2):330–335. 10.1016/j.bbrc.2012.09.055 23000164 10.1016/j.bbrc.2012.09.055

[CR130] Numan M, Guo W, Choi S, Wang X, Du B, Jin W, Bhandari RK, Ligaba‐Osena A (2022) Analysis of miRNAs responsive to long‐term calcium deficiency in tef (*Eragrostis tef* (Zucc.) Trotter). Plant Direct 6(5):e400. 10.1002/pld3.400 35582629 10.1002/pld3.400 PMC9090557

[CR131] Ossowski S, Schwab R, Weigel D (2008) Gene silencing in plants using artificial microRNAs and other small RNAs. Plant J 53(4):674–690. 10.1111/j.1365-313X.2007.03328.x 18269576 10.1111/j.1365-313X.2007.03328.x

[CR132] Palakolanu SR, Gupta S, Yeshvekar RK, Chakravartty N, Kaliamoorthy S, Shankhapal AR, Vempati AS, Kuriakose B, Lekkala SP, Philip M, Perumal RC, Lachagari VBR, Bhatnagar-Mathur P (2022) Genome-wide miRNAs profiles of pearl millet under contrasting high vapor pressure deficit reveal their functional roles in drought stress adaptations. Physiol Plant 174(1):1–17. 10.1111/ppl.13521 10.1111/ppl.13521 34392545

[CR133] Paul S, Datta SK, Datta K (2015) MiRNA regulation of nutrient homeostasis in plants. Front Plant Sci 6:232. 10.3389/fpls.2015.00232 25914709 10.3389/fpls.2015.00232 PMC4392614

[CR134] Paul S, Gayen D, Datta SK, Datta K (2016) Analysis of high iron rice lines reveals new miRNAs that target iron transporters in roots. J Exp Bot 67(19):5811. 10.1093/JXB/ERW346 27729476 10.1093/jxb/erw346 PMC5066498

[CR135] Puli COR, Zheng Y, Li Y-F, Jagadeeswaran G, Suo A, Jiang B, Sharma P, Mann R, Ganesan G, Gogoi N, Srinivasan A, Kakani A, Kakani VG, Barakat A, Sunkar R (2021) MicroRNA profiles in Sorghum exposed to individual drought or heat or their combination. J Plant Biochem Biotechnol 30(4):848–861. 10.1007/s13562-021-00747-0

[CR136] Qiao F, Yang Q, Wang CL, Fan YL, Wu XF, Zhao KJ (2007) Modification of plant height via RNAi suppression of *OsGA20ox2* gene in rice. Euphytica 158(1):35–45. 10.1007/s10681-007-9422-6

[CR137] Rai S, Kaur A, Chopra CS (2018) Gluten-free products for celiac susceptible people. Front Nutr 5:116. 10.3389/fnut.2018.00116 30619866 10.3389/fnut.2018.00116 PMC6304385

[CR138] Raj R, Kumar S, Mishra S, Sherpa D (2025) Development of lysigenous aerenchyma in root alleviates the waterlogging stress in little millet. Trop Plant Biol 18(1):1–12. 10.1007/s12042-025-09439-8

[CR139] Rani V, Rana S, Muthamilarasan M, Joshi DC, Gupta R, Singh R, Yadav D (2025) Identification and characterization of Eco-miR 169-EcNF-YA13 gene regulatory network reveal their role in conferring tolerance to dehydration and salinity stress in finger millet. Sci Rep 15(1):1–23. 10.1038/S41598-025-96233-X;SUBJMETA 40210666 10.1038/s41598-025-96233-x PMC11985966

[CR140] Rashid M, Fahad M, Wu L, Shen Y (2026) Comprehensive genomic and haplotype analysis of the phytocyanin gene family reveals evolutionary expansion and functional diversification in *Setaria italica*. Plant Cell Rep 45(4):102. 10.1007/s00299-026-03783-z 10.1007/s00299-026-03783-z 41874759

[CR141] Ravikesavan R, Sivamurugan AP, Iyanar K, Pramitha JL, Nirmalakumari A (2022) Millet cultivation: An overview. In: Anandharamakrishnan C et al (eds) Handbook of Millets - Processing, Quality, and Nutrition Status. Springer, Singapore. 10.1007/978-981-16-7224-8_2

[CR142] Ray DK, West PC, Clark M, Gerber JS, Prishchepov AV, Chatterjee S (2019) Climate change has likely already affected global food production. PLoS ONE 14(5):e0217148. 10.1371/journal.pone.0217148 31150427 10.1371/journal.pone.0217148 PMC6544233

[CR143] Rodriguez RE, Mecchia MA, Debernardi JM, Schommer C, Weigel D, Palatnik JF (2010) Control of cell proliferation in Arabidopsisthaliana by microRNA miR396. Development 137(1):103–112. 10.1242/dev.043067 10.1242/dev.043067 PMC2796936 20023165

[CR144] Rouached H, Arpat AB, Poirier Y (2010) Regulation of phosphate starvation responses in plants: signaling players and cross-talks. Mol Plant 3(2):288–299. 10.1093/mp/ssp120 20142416 10.1093/mp/ssp120

[CR145] Rurinda J, Mapfumo P, van Wijk MT, Mtambanengwe F, Rufino MC, Chikowo R, Giller KE (2014) Comparative assessment of maize, finger millet and sorghum for household food security in the face of increasing climatic risk. Eur J Agron 55:29–41. 10.1016/j.eja.2013.12.009

[CR146] Saha P, Blumwald E (2016) Spike-dip transformation of *Setaria viridis*. Plant J 86(1):89–101. 10.1111/tpj.13148 26932666 10.1111/tpj.13148

[CR147] Saini RP, Raman V, Dhandapani G, Malhotra EV, Sreevathsa R, Kumar PA, Sharma TR, Pattanayak D (2018) Silencing of HaAce1 gene by host-delivered artificial microRNA disrupts growth and development of *Helicoverpa armigera*. PLoS ONE 13(3):e0194150. 10.1371/journal.pone.0194150 10.1371/journal.pone.0194150 PMC5856398 29547640

[CR148] Saleem MA, Khan A, Tu J, Huang W, Liu Y, Feng N, Zheng D, Xue Y (2025) Salinity stress in rice: multilayered approaches for sustainable tolerance. Int J Mol Sci 26(13):6025. 10.3390/ijms26136025 40649804 10.3390/ijms26136025 PMC12250271

[CR149] Salvador-Guirao R, Baldrich P, Weigel D, Rubio-Somoza I, San Segundo B (2018) The microRNA miR773 is involved in the *Arabidopsis* immune response to fungal pathogens. Mol Plant-Microbe Interact 31(2):249–259. 10.1094/MPMI-05-17-0108-R 10.1094/MPMI-05-17-0108-R 28990488

[CR150] Samad AFA, Sajad M, Nazaruddin N, Fauzi IA, Murad AMA, Zainal Z, Ismail I (2017) MicroRNA and transcription factor: key players in plant regulatory network. Front Plant Sci 8:565. 10.3389/fpls.2017.00565 28446918 10.3389/fpls.2017.00565 PMC5388764

[CR151] Samineni S, Gummadi S, Thushar S, Khan DN, Gkanogiannis A, Becerra Lopez-Lavalle LA, Singh RK (2025) Exploring proso millet resilience to abiotic stresses: high-yield potential in desert environments of the Middle East. Agronomy 15(1):165. 10.3390/agronomy15010165

[CR152] Sanjay Kumar T, Nageswari R, Somasundaram S, Anantharaju P, Baskar M, Ramesh T, Rathika S, Vanniarajan C, Subrahmaniyan K (2024) Significance of millets for food and nutritional security—an overview. Discov Food 4(1):73. 10.1007/s44187-024-00149-w

[CR153] Schwab R, Palatnik JF, Riester M, Schommer C, Schmid M, Weigel D (2005) Specific effects of microRNAs on the plant transcriptome. Dev Cell 8(4):517–527. 10.1016/j.devcel.2005.01.018 15809034 10.1016/j.devcel.2005.01.018

[CR154] Schwab R, Ossowski S, Riester M, Warthmann N, Weigel D (2006) Highly specific gene silencing by artificial microRNAs in *Arabidopsis*. Plant Cell 18(5):1121–1133. 10.1105/tpc.105.039834 16531494 10.1105/tpc.105.039834 PMC1456875

[CR155] Selvam J, Rahman H, Boopathi NM, MR (2015) Computational identification of salinity responsive microRNAs in contrasting genotypes of Finger millet (*Eleusine coracana* L.). Res J Biotechnol 10(9):52–64. 10.1007/s13205-021-02882-6

[CR156] Seo PJ, Ryu J, Kang SK, Park C (2011) Modulation of sugar metabolism by an INDETERMINATE DOMAIN transcription factor contributes to photoperiodic flowering in *Arabidopsis*. Plant J 65(3):418–429. 10.1111/j.1365-313X.2010.04432.x 21265895 10.1111/j.1365-313X.2010.04432.x

[CR157] Sharma R, Sharma S, Dar BN, Singh B (2021) Millets as potential nutri-cereals: a review of nutrient composition, phytochemical profile and techno-functionality. Int J Food Sci Technol 56(8):3703–3718. 10.1111/ijfs.15044

[CR158] Sharma A, Ceasar SA, Pandey H, Devadas VS, Kesavan AK, Heisnam P, Vashishth A, Misra V, Mall AK (2025) Millets: nutrient-rich and climate-resilient crops for sustainable agriculture and diverse culinary applications. J Food Compos Anal 137:106984. 10.1016/j.jfca.2024.106984

[CR159] Shi J, Li R, Qiu D, Jiang C, Long Y, Morgan C, Bancroft I, Zhao J, Meng J (2009) Unraveling the complex trait of crop yield with quantitative trait loci mapping in *Brassica napus*. Genetics 182(3):851–861. 10.1534/genetics.109.101642 10.1534/genetics.109.101642 PMC2710164 19414564

[CR160] Shinde H, Dudhate A, Anand L, Tsugama D, Gupta SK, Liu S, Takano T (2020) Small RNA sequencing reveals the role of pearl millet miRNAs and their targets in salinity stress responses. S Afr J Bot 132:395–402. 10.1016/j.sajb.2020.06.011

[CR161] Shukla P, Aggarwal PR, Choudhary P, Muthamilarasan M (2025) Exploring the nutraceutical potential of foxtail millet: a minor millet with a major impact on nutrition security. Proc Indian Natl Sci Acad 92:52–62. 10.1007/s43538-025-00402-5

[CR162] Simmons T, Styer AB, Pierroz G, Gonçalves AP, Pasricha R, Hazra AB, Bubner P, Coleman-Derr D (2020) Drought drives spatial variation in the millet root microbiome. Front Plant Sci 11:599. 10.3389/fpls.2020.00599 32547572 10.3389/fpls.2020.00599 PMC7270290

[CR163] Singroha G, Sharma P, Sunkur R (2021) Current status of microRNA-mediated regulation of drought stress responses in cereals. Physiol Plant 172(3):1808–1821. 10.1111/ppl.13451 33956991 10.1111/ppl.13451

[CR164] Song JB, Gao S, Sun D, Li H, Shu XX, Yang ZM (2013) miR394 and LCR are involved in *Arabidopsis* salt and drought stress responses in an abscisic acid-dependent manner. BMC Plant Biol 13(1):210. 10.1186/1471-2229-13-210 24330668 10.1186/1471-2229-13-210 PMC3870963

[CR165] Song JB, Gao S, Wang Y, Li BW, Zhang YL, Yang ZM (2016) miR394 and its target gene LCR are involved in cold stress response in *Arabidopsis*. Plant Gene 5:56–64. 10.1016/j.plgene.2015.12.001

[CR166] Song Z, Zhang L, Wang Y, Li H, Li S, Zhao H, Zhang H (2018) Constitutive expression of miR408 improves biomass and seed yield in *Arabidopsis*. Front Plant Sci 8:2114. 10.3389/fpls.2017.02114 10.3389/fpls.2017.02114 PMC5789609 29422907

[CR167] Soto-Suárez M, Baldrich P, Weigel D, Rubio-Somoza I, San Segundo B (2017) The *Arabidopsis* miR396 mediates pathogen-associated molecular pattern-triggered immune responses against fungal pathogens. Sci Rep 7(1):44898. 10.1038/srep44898 28332603 10.1038/srep44898 PMC5362962

[CR168] Stief A, Altmann S, Hoffmann K, Pant BD, Scheible W-R, Bäurle I (2014) *Arabidopsis* miR156 regulates tolerance to recurring environmental stress through SPL transcription factors. Plant Cell 26(4):1792–1807. 10.1105/tpc.114.123851 24769482 10.1105/tpc.114.123851 PMC4036586

[CR169] Sun W, Xu XH, Li Y, Xie L, He Y, Li W, Lu X, Sun H, Xie X (2020) OsmiR530 acts downstream of OsPIL15 to regulate grain yield in rice. New Phytol 226(3):823–837. 10.1111/nph.16399 31883119 10.1111/nph.16399 PMC7187366

[CR170] Sunkar R, Kapoor A, Zhu JK (2006) Erratum: posttranscriptional induction of two Cu/Zn superoxide dismutase genes in *Arabidopsis* is mediated by downregulation of miR398 and important for oxidative stress tolerance. Plant Cell 18(9):2051-2065. 10.1105/tpc.106.180960 10.1105/tpc.106.041673 PMC1533975 16861386

[CR171] Tabassum N, Ahmed HI, Parween S, Sheikh AH, Saad MM, Krattinger SG, Hirt H (2024) Host genotype, soil composition, and geo-climatic factors shape the fonio seed microbiome. Microbiome 12(1):11. 10.1186/s40168-023-01725-5 10.1186/s40168-023-01725-5 PMC10792890 38233870

[CR172] Tanwar R, Panghal A, Chaudhary G, Kumari A, Chhikara N (2023) Nutritional, phytochemical and functional potential of sorghum: a review. Food Chem Adv 3:100501. 10.1016/j.focha.2023.100501

[CR173] Thakur P, Kumar S, Malik JA, Berger JD, Nayyar H (2010) Cold stress effects on reproductive development in grain crops: an overview. Environ Exp Bot 67(3):429–443. 10.1016/j.envexpbot.2009.09.004

[CR174] Tiwari A, Rastogi A, Singh V, Arunachalam A (2020) Effect of water stress on oxidative damage and antioxidant enzyme activity in finger millet and barnyard millet. Indian J Hill Farming 33:36–45. 10.1007/s13205-021-02882-6

[CR175] Tong H, Wang C, Han X, Sun Q, Luo E, Yang C, Xu G, Ou X, Li S, Zhang J, Yang J (2025) Multi-omics-based construction of ncRNA-gene-metabolite networks provides new insights into metabolic regulation under salt stress in rice. Rice 18(1):50. 10.1186/S12284-025-00811-6 40512246 10.1186/s12284-025-00811-6 PMC12165919

[CR176] Tsai W-A, Sung P-H, Kuo Y-W, Chen M-C, Jeng S-T, Lin J-S (2023) Involvement of microRNA164 in responses to heat stress in *Arabidopsis*. Plant Sci 329:111598. 10.1016/j.plantsci.2023.111598 36657663 10.1016/j.plantsci.2023.111598

[CR177] Tyagi S, Kumar A, Gautam T, Pandey R, Rustgi S, Mir RR (2021) Development and use of miRNA-derived SSR markers for the study of genetic diversity, population structure, and characterization of genotypes for breeding heat tolerant wheat varieties. PLoS ONE 16(2):e0231063. 10.1371/JOURNAL.PONE.0231063 33539339 10.1371/journal.pone.0231063 PMC7861453

[CR178] Varshney RK, Sinha P, Singh VK, Kumar A, Zhang Q, Bennetzen JL (2020) 5Gs for crop genetic improvement. Curr Opin Plant Biol 56:190–196. 10.1016/j.pbi.2019.12.004 32005553 10.1016/j.pbi.2019.12.004 PMC7450269

[CR179] Veach AM, Chen H, Yang ZK, Labbe AD, Engle NL, Tschaplinski TJ, Schadt CW, Cregger MA (2020) Plant hosts modify belowground microbial community response to extreme drought. mSystems 5(3):10–1128. 10.1128/mSystems.00092-20 10.1128/mSystems.00092-20 PMC7329318 32606021

[CR180] Vinoth A, Ravindhran R (2017) Biofortification in millets: a sustainable approach for nutritional security. Front Plant Sci 8:29. 10.3389/fpls.2017.00029 28167953 10.3389/fpls.2017.00029 PMC5253353

[CR181] Wang C, Fang J (2015) RLM-RACE, PPM-RACE, and qRT-PCR: an integrated strategy to accurately validate miRNA target genes. In: Rederstorff M (eds) Small Non-Coding RNAs. Methods in Molecular Biology, vol 1296. Humana Press, New York. 10.1007/978-1-4939-2547-6_16 10.1007/978-1-4939-2547-6_16 25791600

[CR182] Wang Y, Li L, Tang S, Liu J, Zhang H, Zhi H, Jia G, Diao X (2016) Combined small RNA and degradome sequencing to identify miRNAs and their targets in response to drought in foxtail millet. BMC Genet 17(1):57. 10.1186/s12863-016-0364-7 27068810 10.1186/s12863-016-0364-7 PMC4828802

[CR183] Wilson ML, VanBuren R (2022) Leveraging millets for developing climate resilient agriculture. Curr Opin Biotechnol 75:102683. 10.1016/j.copbio.2022.102683 35042014 10.1016/j.copbio.2022.102683

[CR184] Wolswinkel P (2024) Long-distance nutrient transport in plants and movement into developing grains. In: Mineral nutrition of crops. CRC Press, Boca Raton. 10.1201/9781003578468

[CR185] Xie K, Shen J, Hou X, Yao J, Li X, Xiao J, Xiong L (2012) Gradual increase of miR156 regulates temporal expression changes of numerous genes during leaf development in rice. Plant Physiol 158(3):1382–1394. 10.1104/pp.111.190488 22271747 10.1104/pp.111.190488 PMC3291253

[CR186] Yadav CB, Muthamilarasan M, Pandey G, Khan Y, Prasad M (2014) Development of novel microRNA-based genetic markers in foxtail millet for genotyping applications in related grass species. Mol Breed 34(4):2219–2224. 10.1007/S11032-014-0137-9/METRICS

[CR187] Yadav T, Kumar A, Yadav RK, Yadav G, Kumar R, Kushwaha M (2020) Salicylic acid and thiourea mitigate the salinity and drought stress on physiological traits governing yield in pearl millet- wheat. Saudi J Biol Sci 27(8):2010–2017. 10.1016/j.sjbs.2020.06.030 32714025 10.1016/j.sjbs.2020.06.030 PMC7376201

[CR188] Yadav OP, Singh DV, Kumari V, Prasad M, Seni S, Singh RK, Sood S, Kant L, Rao BD, Madhusudhana R (2024) Production and cultivation dynamics of millets in India. Crop Sci 64(5):2459–2484. 10.1002/csc2.21207

[CR189] Yang T, Wang Y, Teotia S, Wang Z, Shi C, Sun H, Gu Y, Zhang Z, Tang G (2019) The interaction between miR160 and miR165/166 in the control of leaf development and drought tolerance in *Arabidopsis*. Sci Rep 9(1):2832. 10.1038/s41598-019-39397-7 30808969 10.1038/s41598-019-39397-7 PMC6391385

[CR190] Yang F, Gong X, Zhao P, Cheng L, Zhang Y, Luo Y, Zhu QH, Ye CY (2025) Toward improving multiomic research in understudied cereals. Nat Genet 57(9):2106–2115. 10.1038/s41588-025-02245-8 40670880 10.1038/s41588-025-02245-8

[CR191] Ye CY, Fan L (2021) Orphan crops and their wild relatives in the genomic era. Mol Plant 14(1):27–39. 10.1016/j.molp.2020.12.013 33346062 10.1016/j.molp.2020.12.013

[CR192] Yogindran S, Rajam MV (2021) Host-derived artificial miRNA-mediated silencing of ecdysone receptor gene provides enhanced resistance to *Helicoverpa armigera* in tomato. Genomics 113(1):736–747. 10.1016/j.ygeno.2020.10.004 33058987 10.1016/j.ygeno.2020.10.004

[CR193] Zandalinas SI, Mittler R (2022) Plant responses to multifactorial stress combination. New Phytol 234(4):1161–1167. 10.1111/nph.18087 35278228 10.1111/nph.18087

[CR194] Zhang B (2015) MicroRNA: a new target for improving plant tolerance to abiotic stress. J Exp Bot 66(7):1749–1761. 10.1093/jxb/erv013 25697792 10.1093/jxb/erv013 PMC4669559

[CR195] Zhang LW, Song JB, Shu XX, Zhang Y, Yang ZM (2013a) miR395 is involved in detoxification of cadmium in *Brassica napus*. J Hazard Mater 250:204–211. 10.1016/j.jhazmat.2013.01.053 10.1016/j.jhazmat.2013.01.053 23454459

[CR196] Zhang YC, Yu Y, Wang CY, Li ZY, Liu Q, Xu J, Liao JY, Wang XJ, Qu LH, Chen F (2013b) Overexpression of microRNA OsmiR397 improves rice yield by increasing grain size and promoting panicle branching. Nat Biotechnol 31(9):848. 10.1038/nbt.2646 10.1038/nbt.2646 23873084

[CR197] Zhang JP, Yu Y, Feng YZ, Zhou YF, Zhang F, Yang YW, Lei MQ, Zhang YC, Chen YQ (2017) MiR408 regulates grain yield and photosynthesis via a phytocyanin protein. Plant Physiol 175(3):1175–1185. 10.1104/pp.17.01169 28904074 10.1104/pp.17.01169 PMC5664482

[CR198] Zhang Y, Xiao T, Yi F, Yu J (2023) SimiR396d targets SiGRF1 to regulate drought tolerance and root growth in foxtail millet. Plant Sci 326:111492. 10.1016/j.plantsci.2022.111492 36243168 10.1016/j.plantsci.2022.111492

[CR199] Zhang JP, Liu TS, Zheng J, Jin Z, Zhu Y, Guo JF, Wang GY (2007) Cloning and characterization of a putative 12-oxophytodienoic acid reductase cDNA induced by osmotic stress in roots of foxtail millet. DNA Seq 18(2):138–144. 10.1080/10425170601060764 10.1080/10425170601060764 17364825

[CR200] Zhao M, Tai H, Sun S, Zhang F, Xu Y, Li WX (2012) Cloning and characterization of maize miRNAs involved in responses to nitrogen deficiency. PLoS ONE 7(1):e29669. 10.1371/journal.pone.0029669 22235323 10.1371/journal.pone.0029669 PMC3250470

[CR201] Zhao Y, Xu K, Liu G, Li S, Zhao S, Liu X, Yang X, Xiao K (2020) Global identification and characterization of miRNA family members responsive to potassium deprivation in wheat (*Triticum aestivum* L.). Sci Rep 10(1):15812. 10.1038/s41598-020-72642-y 32978439 10.1038/s41598-020-72642-y PMC7519128

[CR202] Zheng L, Qu L (2015) Application of micro RNA gene resources in the improvement of agronomic traits in rice. Plant Biotechnol J 13(3):329–336. 10.1111/pbi.12321 25583449 10.1111/pbi.12321

[CR203] Zhou J, Deng K, Cheng Y, Zhong Z, Tian LI, Tang XU, Tang A, Zheng X, Zhang T, Qi Y (2017) CRISPR-Cas9 based genome editing reveals new insights into microRNA function and regulation in rice. Front Plant Sci 8:1598. 10.3389/fpls.2017.01598 28955376 10.3389/fpls.2017.01598 PMC5602353

[CR204] Zhou S, Zhu Y, Wang L, Zheng Y, Chen J, Li T, Yang X, Wang H, Li X, Ma X (2020) Osa-miR1873 fine-tunes rice immunity against *Magnaporthe oryzae* and yield traits. J Integr Plant Biol 62(8):1213–1226. 10.1111/jipb.12900 31863525 10.1111/jipb.12900

[CR205] Zhou S, Huang K, Zhou Y, Hu Y, Xiao Y, Chen T, Yin M, Liu Y, Xu M, Jiang X (2022) Degradome sequencing reveals an integrative miRNA-mediated gene interaction network regulating rice seed vigor. BMC Plant Biol 22(1):1–22. 10.1186/S12870-022-03645-2/TABLES/3 35650544 10.1186/s12870-022-03645-2 PMC9158300

[CR206] Zhu Z, Li D, Cong L, Lu X (2021) Identification of microRNAs involved in crosstalk between nitrogen, phosphorus and potassium under multiple nutrient deficiency in sorghum. Crop J 9(2):465–475. 10.1016/j.cj.2020.07.005

[CR207] Zou C, Li L, Miki D, Li D, Tang Q, Xiao L, Rajput S, Deng P, Peng LI, Jia W (2019a) The genome of broomcorn millet. Nat Commun 10(1):436. 10.1038/s41467-019-08409-5 30683860 10.1038/s41467-019-08409-5 PMC6347628

[CR208] Zou J, Huss M, Abid A, Mohammadi P, Torkamani A, Telenti A (2019b) A primer on deep learning in genomics. Nat Genet 51(1):12–18. 10.1038/s41588-018-0295-5 30478442 10.1038/s41588-018-0295-5 PMC11180539

[CR209] Zubair M, Khan MZ, Rauf I, Raza A, Shah AH, Hassan I, Amin I, Mansoor S (2020) Artificial micro RNA (amiRNA)-mediated resistance against whitefly (*Bemisia tabaci*) targeting three genes. Crop Prot 137:105308. 10.1016/j.cropro.2020.105308

